# WRKY genes family study reveals tissue-specific and stress-responsive TFs in wild potato species

**DOI:** 10.1038/s41598-020-63823-w

**Published:** 2020-04-28

**Authors:** Clizia Villano, Salvatore Esposito, Vincenzo D’Amelia, Raffaele Garramone, Daniela Alioto, Astolfo Zoina, Riccardo Aversano, Domenico Carputo

**Affiliations:** 1 Department of Agricultural Sciences, University of Naples Federico II, via Università 100, 80055 Portici, Italy; 2 CREA Via Cavalleggeri 25, 84098 Pontecagnano-Faiano, Italy; 3 National Research Council of Italy, Institute of Biosciences and Bioresources (CNR-IBBR), Via Università 133, Portici, NA Italy; 4 IPSP-CNR, Via Università 133, 80055 Portici, Italy

**Keywords:** Plant sciences, Natural variation in plants

## Abstract

Wild potatoes, as dynamic resource adapted to various environmental conditions, represent a powerful and informative reservoir of genes useful for breeding efforts. WRKY transcription factors (TFs) are encoded by one of the largest families in plants and are involved in several biological processes such as growth and development, signal transduction, and plant defence against stress. In this study, 79 and 84 genes encoding putative WRKY TFs have been identified in two wild potato relatives, *Solanum commersonii* and *S. chacoense*. Phylogenetic analysis of WRKY proteins divided *ScWRKYs* and *SchWRKYs* into three Groups and seven subGroups. Structural and phylogenetic comparative analyses suggested an interspecific variability of WRKYs. Analysis of gene expression profiles in different tissues and under various stresses allowed to select *ScWRKY045* as a good candidate in wounding-response, *ScWRKY055* as a bacterial infection triggered *WRKY* and *ScWRKY023* as a multiple stress-responsive *WRKY* gene. Those *WRKYs* were further studied through interactome analysis allowing the identification of potential co-expression relationships between *ScWRKYs*/*SchWRKYs* and genes of various pathways. Overall, this study enabled the discrimination of *WRKY* genes that could be considered as potential candidates in both breeding programs and functional studies.

## Introduction

Plants experience environmental constrains and pathogen attacks during their life. Being sessile organisms, their survival depends on the ability to properly and promptly reprogram cellular networks. Several and different classes of transcription factors (TFs) work as “master regulators” and “selector genes”, being able to control processes that specify cell types and developmental patterning and modulate specific pathways. Among them, WRKY factors are drawing a great deal of interest in the scientific community due to their ability to simultaneously cope with multiple stresses^1,2^. They are notorious for coordinating signals in plant immunity response against several pathogens and pest attacks^3,4^. More recently, it has been confirmed that WRKYs also base defence mechanism to abiotic stresses and play a key role in cross-talk pathway networks between plant response and development^5,6^. Their involvement into multiple stress response and in plant growth regulation is evidenced by their W-box specific DNA binding^7,8^. Besides, WRKY binds sugar responsive elements and, very recently, it has been demonstrated that they activate sugar responsive genes through an epigenetic mechanism of control^9^. The systematic classification of components of the WRKY family is well organized. It is based on the WRKY binding domain (WD) characteristics along with those of the Zinc Finger (ZF) motif, which is typically present downstream the WD. WD consists of 60 amino acids structured as four-stranded β-sheets able to enter the major groove of B-form DNA. The highly conserved motif is “WRKYGQK”. According to the number of WDs and the type of zinc finger motif, WRKY proteins can be classified into three Groups, namely Group I, II, and III: Group I WRKY members contain two WDs with two classical C_2_H_2_ ZF motifs, Group II WRKYs have one WD with one C_2_H_2_ ZF motif, and Group III WRKYs contain one WD with one C_2_HC ZF motif ^3,5^. Group II WRKYs can be divided into five subGroups (IIa-IIe)^10^. It is well recognized that Group I WRKY members are the evolutionary ancestors of the other WRKYs and that they exist only in lower plants^11,12^. The complexity of this gene family involves different molecular levels, from the transcriptional self-regulation through microRNAs to post-transcriptional events, such as alternative splicing, post-translational regulation through ubiquitin proteasome system and MAPK cascade^9^. Studies addressed to mine sequence divergences or to identify gene expression differences in WRKYs of cultivated and wild species are increasing. Such investigations may pave the way into exploiting these regulators for breeding purposes. A recent study carried out in the sweet potato wild ancestor *Ipomoea trifida*, highlighted how investigations on WRKY gene family in wild relatives can boost the molecular breeding of cultivated species^13^. However, our knowledge is still not complete and therefore WRKY gene biodiversity remains unlocked in many species.

The potato, *Solanum tuberosum*, is one of the most cultivated non-cereal crop in the world. Its cultivation is often hampered by the fact that it is susceptible to a wide range of stressors causing severe yield losses. Sources of resistance can be found in its tuber-bearing wild relatives, that are highly used as rootstock for cultivated Solanaceae^14^ but poorly used in breeding programs. However, recent technologies can be implemented to enhance this precious source of genes/alleles. Among them, genome sequences are opening new paths for both basic research and varietal development. Nowadays, the genome sequence of two wild potato species, *S. commersonii* and *S. chacoense*, are available^15,16^. These species are excellent sources of tolerance to both biotic stressors, such as *Ralstonia solanacearum*^17^, *Phytophthora infestans*^18^ and *Pectobacterium carotovorum*^19^, and abiotic constraints, such as cold^15^ and drought^20^. Despite this, to date no studies have examined WRKY gene family components and their different characteristics in wild potato species. A few data on this gene family are available only in the cultivated potato, where Zhang *et al*.^21^, Liu *et al*.^22^ and Cheng *et al*.^12^ identified 79, 82 and 81 *StWRKYs*, respectively. Previously, Dellagi *et al*.^23^ identified *StWRKY1* as a good candidate for functional studies, and Shahzad *et al*.^24^ overexpressed it in potato. They provided evidence that *StWRKY1* acts as positive regulator of biotic and abiotic stress resistance through the activation of basal defence networks. Here, for the first time, we report a detailed analysis of WRKY genes in the genome of *S. commersonii* and *S. chacoense*, providing subGroup classification, gene structure and conserved motif composition. We analysed the patterns of *ScWRKYs* and *SchWRKYs* expression in flowers, leaves and tubers to determine whether some WRKYs own tissue-specificity. Furthermore, we used *S. commersonii* to highlight expression changes of selected *ScWRKY* genes after wounding and biotic (Potato Virus Y and *P. carotovorum*) stresses. Through the data here presented, the work aims to give a picture of the potato wild WRKY members, their nature and the complexity of their responses to unfavourable situations.

## Materials and Methods

### Identification of WRKY in S. commersonii and S. chacoense and phylogenetic analysis

The well-known WRKY protein sequences of *S. tuberosum*^22^ and *A. thaliana*^25^ were used as queries to build an HMM profile through HMMER as reported by Esposito *et al.*^26^ and to search orthologs in *S. commersonii* (cmm1T clone of PI243503) and *S. chacoense* (M6 clone) genomes. Only sequences with an e-value lower than 10^−5^ and an identity higher than 55% were regarded as putative WRKYs and further analyzed. The full-lenght WRKY candidate proteins were then manually confirmed by checking the WRKY domain using the NCBI search domain online tool^26^ and used for the phylogenetic analysis. Names were assigned based on *S. tuberosum* orthologs using bootstrap replicates of the Maximum Likelihood (ML) phylogenetic tree (values higher than 50). Briefly, MEGAX^27^ was first used to establish the best-fit model of evolution through the option “Find best DNA/Protein Models” implemented in the program and then for phylogenetic tree building using the appropriate options. In the phylogenetic analysis were integrated seven AtWRKY proteins randomly selected as representative of each WRKY Group, as already reported by Karanja *et al*.^28^. One-to-one orthologs were considered when candidate proteins allocated on the same clade in the phylogenetic tree with *S. tuberosum*. The exon-intron organization of WRKY genes was determined using the online GSDS tool (http://gsds.cbi.pku.edu.cn). Finally, the on-line tool Phenogram (http://visualization.ritchielab.org/phenograms/plot) was used to determine the location of the WRKY genes on *S. chacoense* chromosomes.

### Public RNAseq-based expression analysis

The transcriptional activity of WRKY genes related to three tissues (flower, leaf and tuber) in *S. commersonii* and *S. chacoense* was estimated using the publicly available RNAseq data sets. As far as *S. commersonii* is concerned, we used raw single-end fastq data deposited under study SRP050412. Briefly, to remove unwanted sequences originating from organelles, reads were mapped against the mitochondrial (*S_tuberosum*_Group_Phureja_ mitochondrion_DM1-3-516-R44) and chloroplast (*S._tuberosum*_Group_Phureja_chloroplast_DM1-3-516-R44) genomes using BOWTIE2 2.2.2^29^ with sensitive local mapping. Unmapped reads were mapped against the *S. commersonii* genome. The BAM files were then analyzed using Cufflinks–Cuffquant software (version 2.2.1) to assemble the aligned reads and to access transcriptome complexity. Expression values for each gene were estimated based on RPKM (Reads Per Kilobase of transcript per Million mapped reads) using the default options. No biological replicates were available for *S. commersonii*. As for *S. chacoense*, data were expressed as mean of biological replicates and RPKM values we directly retrieved from SpudDB (http://solanaceae.plantbiology.msu.edu). For all StWRKY orthologs we recovered from the public *S. tuberosum* database (http://solanaceae.plantbiology.msu.edu) transcriptional data regarding potato leaves subjected to salt stress (50 mM NaCl for 24 h), osmotic stress (260 μM mannitol for 24 h), heat stress (35 °C for 24 h) and treatments with 6-benzylaminopurine (BAP) (10 µM for 24 h), abscisic acid (ABA) (50 µM for 24 h), indole-3-acetic acid (IAA) (10 µM for 24 h), gibberellic acid (GA3) (50 µM for 24 h), β-aminobutyric acid (BABA) (24, 48, 72 h), benzothiadiazole (BTH) (24, 48, 72 h), and *invitro* culture (root and shoot).

### Plant materials and stress treatments

*In-vitro* plantlets of *S. commersonii* clone cmm1T, accession PI243503, derived from the Inter-Regional Potato Introduction Station (Sturgeon Bay, Wisconsin), were micro-propagated as described by D’Amelia *et al*.^30^. Four-week-old vitroplants were transplanted into 14-mm plastic pots containing sterile soil and grown in a greenhouse under long-day conditions (16-h light, 8-h dark); temperature was set at 26 °C during the day and 18 °C at night. Three-week-old seedlings were used for all stress experiments and sampled in a 0, 1, 2, 4, 6 hpt (hours post treatment) time course. As for virus infection, young plants of clone cmm1T were mechanically inoculated with Potato Virus Y tuber necrotic strain (PVY^NTN^) as reported by Esposito *et al*.^31^. For assessing bacterial resistance, the protocol of Melito *et al*.^32^ was used with few modifications. The stem base of vitroplants (one injection per plant) was inoculated with 20 μl of *P. carotovorum* strain Ecc 009 sospension under greenhouse conditions (with temperatures ranging from 20 to 30 °C during the day and from 12 to 17 °C during the night). The bacterial culture was adjusted to 10^6^ CFU·mL^−1^ in MgCl_2_ solution. The whole plant was then covered with a transparent plastic bag. For both treatments (viral and bacterial), plants inoculated with buffer were considered as mock control. At each time point, leaves were collected from three biological replicates, both for treated and untreated samples. Each biological replicate consisted of a pool of three plants. Young leaf samples were collected from treated and mock control plants following the time course and stored at − 80 °C before RNA extraction. Wounding stress was induced according to the protocol of Vannozzi *et al*.^33^ with few modifications. Leaf discs (15 mm diameter) were punched from healthy leaves detached from glasshouse-grown plantlets and incubated upside down on 3MM moist filter paper in large Petri dishes at 22 °C under 12 h light / 12 h dark conditions until harvest. Collected discs were immediately frozen in liquid nitrogen and stored at −80 °C for subsequent RNA extraction. Five discs were randomly chosen per each time point. No treated leaves were used as control. Each treatment consisted of three biological replications.

### RNA extraction, cDNA synthesis and quantitative Real-Time PCR (RT-qPCR)

Total RNA was isolated from 100 mg of grinded leaves as reported by Rinaldi *et al*.^34^ and Villano *et al*.^35^. The Spectrum^TM^ Plant Total RNA kit (Sigma-Aldrich, St. Louis, MO, USA) was used following the manufacturer’s protocol with some modifications. Quantity and quality of the isolated RNA was measured using the NanoDrop ND-1000 spectrophotometer (Thermo Scientific, Wilmington, DE, USA) and Qubit 2.0 fluorometer (Life Technologies, Carlsbad, CA). For cDNA synthesis, 1 µg of each RNA sample was reverse transcribed using the SuperScript III cDNA Synthesis Kit (Life Technologies, Paisley, UK) following the manufacturer’s protocol. Specific primers were designed using the website Primer3 as reported by Koressaar *et al*.^36^ (Supplementary Table 1). Expression analysis was conducted by RT-qPCR as reported by Di Meo *et al*.^37^ and Brulè *et al*.^38^ using a SYBR Green method on a 7900HT Fast Real-Time PCR System (Applied Biosystems, Foster City, CA, USA). Each 15 µL PCR reaction contained 330 nM of each primer, 2 µL of 5-fold diluted cDNA and 7.5 µL of SYBR Green Mix (Applied Biosystems, Foster City, CA, USA). The SDS 2.3 and RQ Manager 1.2 software (both Applied Biosystems, Foster City, CA, USA) were used for data elaboration. The expression of each target gene was normalized with the expression level of the housekeeping gene (Elongation Factor) and calibrated with the mock control using the Livak method, obtaining the values in log_2_(FC)^39^. Each analysis consisted of three technical replications.

### Protein-protein interaction in silico analyses

An interactome analysis was carried out to investigate the function of tissue-specific and stress responsive *ScWRKYs* and *SchWRKYs* selected in the expression study through the analysis of direct ortholog of *StWRKY* genes. The protein-protein interaction networks STRING database was used (http://string-db.org/). It reports protein associations based on various sources, such as experimental results, pathway understanding, text-mining and genomic information^40^. The interactome was constructed using a medium confidence score (0.400).

## Results

### Phylogenetic analysis and classification of ScWRKYs and SchWRKYs

**Figure 1:**
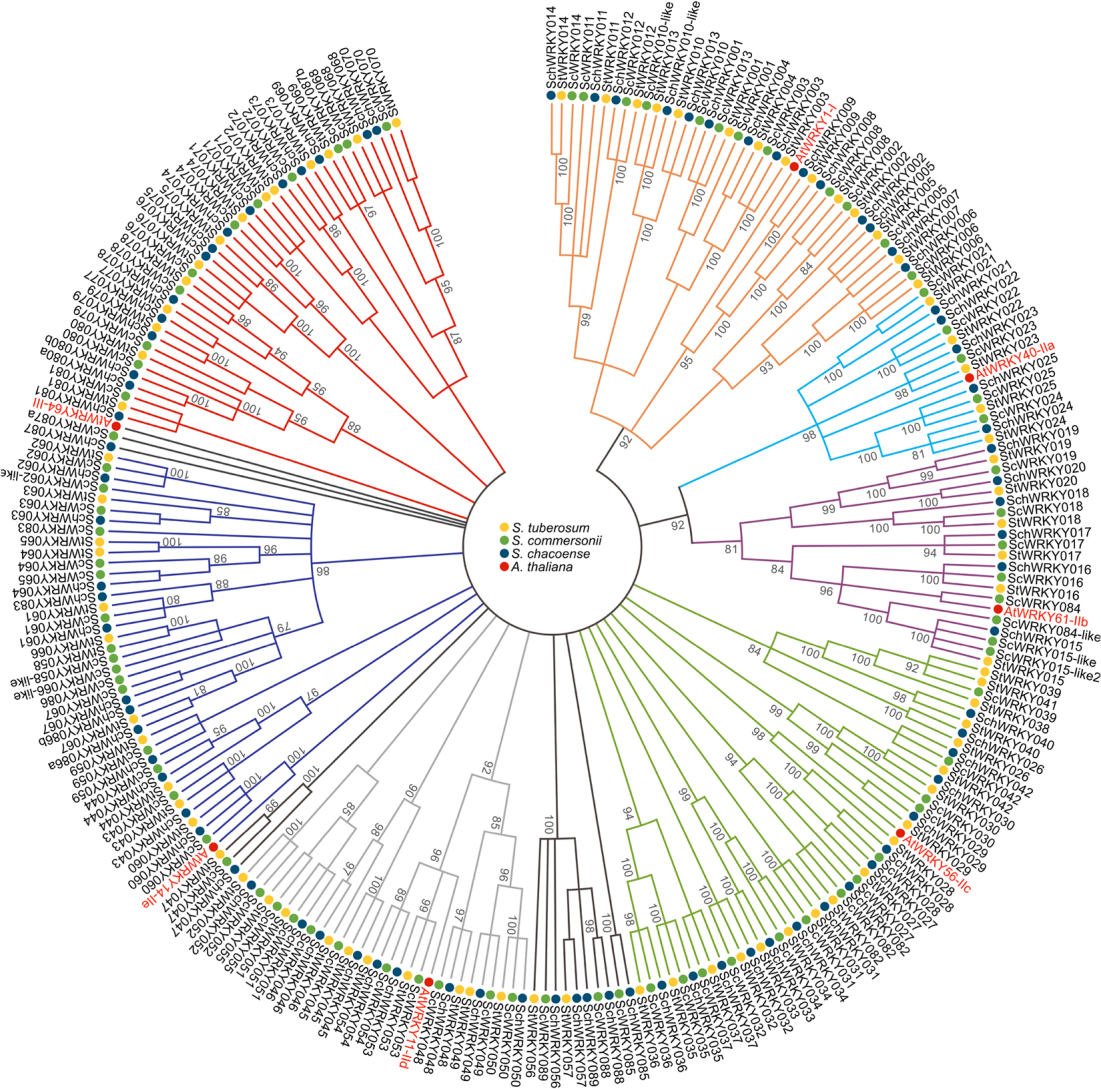
Phylogenetic analysis WRKY proteins in *S. commersonii*, *S. chacoense* and seven representative proteins of *Arabidopsis*. Multiple sequence alignments of WRKY amino acid sequences were performed using ClustalX, and the phylogenetic tree was constructed using MEGAX by the Maximum Likelihood (ML) method and 1000 bootstrap replicates. The tree was divided into seven phylogenetic subGroups and distinguished by colours: dark purple for Group I, light blue for subGroup IIa, orange for subGroup IIb, light purple for subGroup IIc, dark blue for subGroup IId, green for subGroup IIe, red for Group III. The bootstrap values were ≥85%.

A total of 79 and 84 candidates corresponding to the Pfam WRKY family were distinguished in *S. commersonii* and *S. chacoense*, respectively (Table 1). Based on phylogenetic analysis, 71 *ScWRKYs* and 80 in *SchWRKYs* were identified as direct orthologs of *StWRKYs*, while the remaining were classified as not direct orthologs and named with the suffix -*like*. Two paralog genes of *SchWRKY080, SchWRKY086* and *ScWRKY087* were also identified and named with the suffix -a and -b (Table 1). The phylogenetic analysis of seven AtWRKY proteins randomly selected as representative of each WRKY Group and all *S. commersonii* and *S. chacoense* WRKY proteins revealed *ScWRKY* and *SchWRKY* classification in three large Groups corresponding to Group I, II and III (Fig. 1), with the exception of nine proteins in *S. commersonii* (*ScWRKY047,ScWRKY051, ScWRKY052, ScWRKY055, ScWRKY085, ScWRKY087a*, *ScWRKY087b, ScWRKY088* and *ScWRKY089*) and eight proteins in *S. chacoense* (*SchWRKY047, SchWRKY051, SchWRKY052, SchWRKY056, SchWRKY057, SchWRKY085, SchWRKY088* and *SchWRKY089*), that were not assigned to any Group (Table 1). In *S. commersonii*, 12 ScWRKY proteins belonged to Group I, 47 to Group II, and 10 to Group III. Group II proteins were further categorized into subGroups. Group IIa, IIb, IIc, IId and IIe included 5, 8, 13, 7 and 14 ScWRKYs respectively (Table 1). As far as *S. chacoense* is concerned, 14 proteins belonging to Group I, 45 to Group II, and 15 to Group III were identified. Those of Group II were classified in subGroup IIa (5 SchWRKYs), IIb (5), IIc (15), IId (7) and IIe (12) (Table 1). Gene and protein features, including the length of the protein sequence, the WRKY domain motif composition and the exons/introns number were analyzed and reported in Supplementary Table 2. In *S. commersonii*, the “WRKYGQK” pattern was highly conserved in 69 ScWRKYs, while five variations were observed in the other proteins (“WGKYGQK”, “WRWLKCG”, “WSKYGQK”, “WRKCGQK”, “WRKYGMK”). In *S. chacoense*, 74 SchWRKYs contained the “WRKYGQK” domain, while the other proteins contained one of the following variations: “WIKYGEN”, “WHKYGQK”, “WRKYGMK”, “WKKHGSN”, “WHKCGQK”. Concerning the Zinc Finger motif, the most common pattern in both species was “C-X_4-5-7_-C-X_22-23-24_-H-X-H/C”. The only exceptions were *ScWRKY068* with “C-X_8_-C-X_27_-H-X_2_-H”, *ScWRKY074* with “C-X_1_-C-X_26_-H-X-C”, and *SchWRKY074* with “C-X_8_-C-X_24_-H-X-C”. Regarding the number of WDs in the studied proteins, out of 12 members belonging to Group I in *S. commersonii*, eight contained two WDs, two had two WDs and other two possessed three WDs. All Group I members in *S. chacoense* harbored two WDs, except SchWRKY014 (one WD). Seven ScWRKYs belonging to Group II and two of Group III contained two WDs, while all other members had only one WD. In *S. chacoense*, Group II and III proteins harbored one WD. All Group III members contained the H_X_C Zinc Finger domain (Supplementary Table 2). Our analysis pointed out that the number of amino acids of ScWRKYs varied from 107 (*ScWRKY30*) to 752 (*ScWRKY87*), and that of *SchWRKYs* from 123 (*SchWRKY21)* to 744 (*SchWRKY3)* (Supplementary Table 2). The exon-intron organization of our *WRKY* genes was examined to gain more insight into the evolution of the *WRKY* family in potato. As shown in Supplementary Table 2, all *ScWRKY* genes possessed from one to eight exons. A similar trend was observed in *S. chacoense*. Concerning the genomic localization of WRKY genes, due to the unavailability of *S. commersonii* physical map, we plotted genes only on *S. chacoense* chromosomes using the Phenogram on-line tool (http://visualization.ritchielab.org/phenograms/plot) (Figure S1). Out of 84 *SchWRKY* gene*s* identified, 83 were mapped. As represented in Figure S1, most of the genes were located on chromosome 3 (11 genes; 13.1%), followed by chromosome 5 (10; 11.9%), Unknown (8; 9.5%) and 2 (7; 8.3%). A total of 25 *SchWRKY* genes (5 on each chromosome) were localized on chromosomes 7 to 12, whereas no one was mapped on chromosome 11.

**Table 1:** List of *ScWRKYs* and *SchWRKYs* with the locus ID and the division in Groups.

S. tuberosum WRKYs	S. commersonii WRKYs	Locus ID ScWRKYs	ScWRKY Groups	S. chacoense WRKYs	Locus ID SchWRKYs	SchWRKY Groups
StWRKY001	ScWRKY001	maker_scaffold1882_snap_gene_0_38_mRNA_1	I	SchWRKY001	g8177.t1	I
StWRKY002	ScWRKY002	maker_scaffold7854_augustus_gene_0_54_mRNA_1	I	SchWRKY002	g13037.t1	I
StWRKY003	ScWRKY003	maker_scaffold2503_augustus_gene_0_43_mRNA_1	I	SchWRKY003	g1652.t1	I
StWRKY004	-	-		SchWRKY004	g27614.t1	I
StWRKY005	ScWRKY005	maker_scaffold31249_augustus_gene_0_94_mRNA_1	I	SchWRKY005	g9868.t1	I
StWRKY006	ScWRKY006	augustus_masked_scaffold354_abinit_gene_0_10_mRNA_1	I	SchWRKY006	g2882.t1	I
StWRKY007	-	-		SchWRKY007	g5502.t1	I
StWRKY008	ScWRKY008	maker_scaffold9215_augustus_gene_0_73_mRNA_1	I	SchWRKY008	g35580.t1	I
StWRKY009	-	-		SchWRKY009	g34576.t1	I
StWRKY010	ScWRKY010	maker_scaffold440_augustus_gene_0_51_mRNA_1	I	-	-	
-	ScWRKY010-like	maker_scaffold5761_snap_gene_0_26_mRNA_1	I	SchWRKY010-like	g35137.t1	I
StWRKY011	ScWRKY011	maker_scaffold1729_augustus_gene_0_61_mRNA_1	I	SchWRKY011	g18246.t1	I
StWRKY012	ScWRKY012	genemark_scaffold41213_abinit_gene_0_8_mRNA_1	I	SchWRKY012	g1746.t1	I
StWRKY013	ScWRKY013	genemark_scaffold21247_abinit_gene_0_14_mRNA_1	I	SchWRKY013	g22208.t1	I
StWRKY014	ScWRKY014	augustus_masked_scaffold89_abinit_gene_0_4_mRNA_1	I	SchWRKY014	g31999.t1	I
StWRKY015	-	-		SchWRKY015	g31090.t1	IIb
-	ScWRKY015-like	augustus_masked_scaffold10618_abinit_gene_0_2_mRNA_1	IIb	-	-	
-	ScWRKY015-like_2	maker_scaffold5837_augustus_gene_0_23_mRNA_1	IIb	-	-	
StWRKY016	ScWRKY016	maker_scaffold13177_augustus_gene_0_11_mRNA_1	IIb	SchWRKY016	g27534.t1	IIb
StWRKY017	ScWRKY017	maker_scaffold17033_augustus_gene_1_25_mRNA_1	IIb	SchWRKY017	g9538.t1	IIb
StWRKY018	ScWRKY018	maker_scaffold24758_augustus_gene_0_43_mRNA_1	IIb	SchWRKY018	g30842.t1	IIb
StWRKY019	ScWRKY019	maker_scaffold11314_augustus_gene_0_23_mRNA_1	IIb	SchWRKY019	g8360.t1	IIb
StWRKY020	-	-		SchWRKY020	g2454.t1	IIb
StWRKY021	ScWRKY021	maker_scaffold31159_snap_gene_0_73_mRNA_1	IIa	SchWRKY021	g11533.t1	IIa
StWRKY022	ScWRKY022	maker_scaffold2968_augustus_gene_0_60_mRNA_1	IIa	SchWRKY022	g16975.t1	IIa
StWRKY023	ScWRKY023	maker_scaffold9305_augustus_gene_0_17_mRNA_1	IIa	SchWRKY023	g5351.t1	IIa
StWRKY024	ScWRKY024	maker_scaffold27786_augustus_gene_0_38_mRNA_1	IIa	SchWRKY024	g31307.t1	IIa
StWRKY025	ScWRKY025	maker_scaffold38372_augustus_gene_0_21_mRNA_1	IIa	SchWRKY025	g31308.t1	IIa
StWRKY026	-	-		SchWRKY026	g32923.t1	IIc
StWRKY027	ScWRKY027	augustus_masked_scaffold4984_abinit_gene_0_0_mRNA_1	IIc	SchWRKY027	g36153.t1	IIc
StWRKY028	ScWRKY028	augustus_masked_scaffold1174_abinit_gene_0_6_mRNA_1	IIc	SchWRKY028	g15762.t1	IIc
StWRKY029	ScWRKY029	maker_scaffold7139_snap_gene_1_53_mRNA_1	IIc	SchWRKY029	g14398.t1	IIc
StWRKY030	ScWRKY030	augustus_masked_scaffold7796_abinit_gene_0_0_mRNA_1	IIc	SchWRKY030	g20531.t1	IIc
StWRKY031	ScWRKY031	maker_scaffold1339_augustus_gene_0_64_mRNA_1	IIc	SchWRKY031	g5592.t1	IIc
StWRKY032	ScWRKY032	maker_scaffold8864_snap_gene_1_45_mRNA_1	IIc	SchWRKY032	9895.t1	IIc
StWRKY033	-	-		SchWRKY033	g11063.t1	IIc
StWRKY034	ScWRKY034	genemark_scaffold11173_abinit_gene_0_31_mRNA_1	IIc	SchWRKY034	g10072.t1	IIc
StWRKY035	ScWRKY035	maker_scaffold23185_snap_gene_0_11_mRNA_1	IIc	SchWRKY035	g20677.t1	IIc
StWRKY036	ScWRKY036	maker_scaffold7687_snap_gene_0_74_mRNA_1	IIc	SchWRKY036	g9069.t1	IIc
StWRKY037	ScWRKY037	maker_scaffold230_augustus_gene_0_11_mRNA_1	IIc	SchWRKY037	g1078.t1	IIc
StWRKY038	-	-		-	-	
StWRKY039	ScWRKY039	maker_scaffold24623_snap_gene_0_85_mRNA_1	IIc	-	-	
StWRKY040	-	-		SchWRKY040	g17334.t1	IIc
StWRKY041	-	-		-	-	
StWRKY042	ScWRKY042	maker_scaffold3388_augustus_gene_0_57_mRNA_1	IIc	SchWRKY042	g39518.t1	IIc
StWRKY043	ScWRKY043	maker_scaffold3609_augustus_gene_0_44_mRNA_1	IIe	SchWRKY043	g28733.t1	IIe
StWRKY044	ScWRKY044	maker_scaffold9525_snap_gene_0_21_mRNA_1	IIe	SchWRKY044	g4268.t1	IIe
StWRKY045	ScWRKY045	maker_scaffold3826_snap_gene_0_9_mRNA_1	IId	SchWRKY045	g37677.t1	IId
StWRKY046	ScWRKY046	maker_scaffold36167_augustus_gene_0_25_mRNA_1	IId	SchWRKY046	g15556.t1	IId
StWRKY047	ScWRKY047	maker_scaffold13022_augustus_gene_0_10_mRNA_1	n.a.	SchWRKY047	g5604.t1	n.a.
StWRKY048	ScWRKY048	maker_scaffold13210_augustus_gene_0_60_mRNA_1	IId	SchWRKY048	g25995.t1	IId
StWRKY049	ScWRKY049	maker_scaffold20941_augustus_gene_0_53_mRNA_1	IId	SchWRKY049	g19483.t1	IId
StWRKY050	ScWRKY050	maker_scaffold37089_augustus_gene_0_11_mRNA_1	IId	SchWRKY050	g33267.t1	IId
StWRKY051	ScWRKY051	maker_scaffold23049_snap_gene_0_12_mRNA_1	n.a.	SchWRKY051	g25365.t1	n.a.
StWRKY052	ScWRKY052	maker_scaffold10802_augustus_gene_0_67_mRNA_1	n.a.	SchWRKY052	g17075.t1	n.a.
StWRKY053	ScWRKY053	maker_scaffold27282_augustus_gene_1_73_mRNA_1	IId	SchWRKY053	g3467.t1	IId
StWRKY054	ScWRKY054	maker_scaffold16944_snap_gene_0_17_mRNA_1	IId	SchWRKY054	g22375.t1	IId
StWRKY055	ScWRKY055	maker_scaffold15104_snap_gene_0_20_mRNA_1	n.a.	-	-	
StWRKY056	-	-		SchWRKY056	g9378.t1	n.a.
StWRKY057	-	-		SchWRKY057	g9376.t1	n.a.
StWRKY058	ScWRKY058-like	maker_scaffold19913_augustus_gene_0_9_mRNA_1	IIe	-	-	
StWRKY059	ScWRKY059	maker_scaffold1174_augustus_gene_0_58_mRNA_1	IIe	SchWRKY059	g15764.t1	IIe
StWRKY060	ScWRKY060	augustus_masked_scaffold18408_abinit_gene_0_2_mRNA_1	IIe	SchWRKY060	g30025.t1	IIe
StWRKY061	ScWRKY061	genemark_scaffold32401_abinit_gene_0_3_mRNA_1	IIe	SchWRKY061	g24860.t1	IIe
StWRKY062	ScWRKY062	augustus_masked_scaffold5103_abinit_gene_0_3_mRNA_1	IIe	SchWRKY062	g9225.t1	IIe
-	ScWRKY062-like	maker_scaffold1081_snap_gene_0_34_mRNA_1	IIe	-	-	
StWRKY063	ScWRKY063	maker_scaffold1081_snap_gene_0_35_mRNA_1	IIe	SchWRKY063	g9218.t1	IIe
StWRKY064	ScWRKY064	maker_scaffold17826_snap_gene_0_36_mRNA_1_like	IIe	SchWRKY064	g32306.t1	IIe
StWRKY065	ScWRKY065	genemark_scaffold17826_abinit_gene_0_25_mRNA_1_like	IIe	-	-	
StWRKY066	ScWRKY066-like	maker_scaffold35381_snap_gene_0_8_mRNA_1	IIe	-	-	
StWRKY067	ScWRKY067	maker_scaffold1552_snap_gene_0_60_mRNA_1	IIe	SchWRKY067	g9511.t1	IIe
StWRKY068	ScWRKY068	genemark_scaffold25887_abinit_gene_0_18_mRNA_1	III	SchWRKY068	g21153.t1	III
StWRKY069	-	-		SchWRKY069	g24755.t1	III
StWRKY070	ScWRKY070	maker_scaffold30616_augustus_gene_0_60_mRNA_1	III	SchWRKY070	g13833.t1	III
StWRKY071	ScWRKY071	maker_scaffold12441_snap_gene_0_28_mRNA_1	III	SchWRKY071	g30258.t1	III
StWRKY072	ScWRKY072	maker_scaffold12583_augustus_gene_0_32_mRNA_1	III	SchWRKY072	g4219.t1	III
StWRKY073	-	-		SchWRKY073	g9466.t1	III
StWRKY074	ScWRKY074	maker_scaffold31861_augustus_gene_0_50_mRNA_1	III	SchWRKY074	g6934.t1	III
StWRKY075	ScWRKY075	-		SchWRKY075	g6933.t1	III
StWRKY076	ScWRKY076	snap_masked_scaffold31861_abinit_gene_0_36_mRNA_1	III	SchWRKY076	g6930.t1	III
StWRKY077	-	-		SchWRKY077	g3563.t1	III
StWRKY078	ScWRKY078	maker_scaffold978_augustus_gene_1_25_mRNA_1	III	SchWRKY078	g22449.t1	III
StWRKY079	ScWRKY079	maker_scaffold3600_augustus_gene_0_37_mRNA_1	III	SchWRKY079	g32230.t1	III
StWRKY080	ScWRKY080	maker_scaffold15162_snap_gene_0_46_mRNA_1	III	SchWRKY080a	novel_model_169_57a387f8	III
-	-	-		SchWRKY080b	temp_model_12.1.57a38f05	III
StWRKY081	ScWRKY081	maker_scaffold7208_snap_gene_0_38_mRNA_1	III	SchWRKY081	g3537.t1	III
StWRKY082	ScWRKY082	augustus_masked_scaffold568_abinit_gene_0_2_mRNA_1	IIc	SchWRKY082	g37078.t1	IIc
-	ScWRKY083	augustus_masked_scaffold6878_abinit_gene_0_0_mRNA_1	IIe	SchWRKY083	g15604.t1	IIe
-	ScWRKY084	augustus_masked_scaffold10960_abinit_gene_0_3_mRNA_1	IIa		-	
-	ScWRKY084_like	maker_scaffold5413_augustus_gene_0_50_mRNA_1	IIb		-	
-	ScWRKY085	augustus_masked_scaffold12000_abinit_gene_0_1_mRNA_1	IIb	SchWRKY085	g10699.t1	n.a.
-	-	-		SchWRKY086a	g9512.t1	IIe
-	-	-		SchWRKY086b	g9513.t1	IIe
-	ScWRKY087a	maker_scaffold2503_augustus_gene_0_39_mRNA_1	n.a.	-	-	
-	ScWRKY087b	snap_masked_scaffold25887_abinit_gene_0_23_mRNA_1	n.a.	-	-	
-	ScWRKY088	maker_scaffold12465_snap_gene_0_41_mRNA_1	n.a.	SchWRKY088	g9374.t1	n.a.
-	ScWRKY089	maker_scaffold42117_snap_gene_0_27_mRNA_1	n.a.	SchWRKY089	g9372.t1	n.a.

### Expression patterns of WRKY genes in S. commersonii and S. chacoense

**Figure 2:**
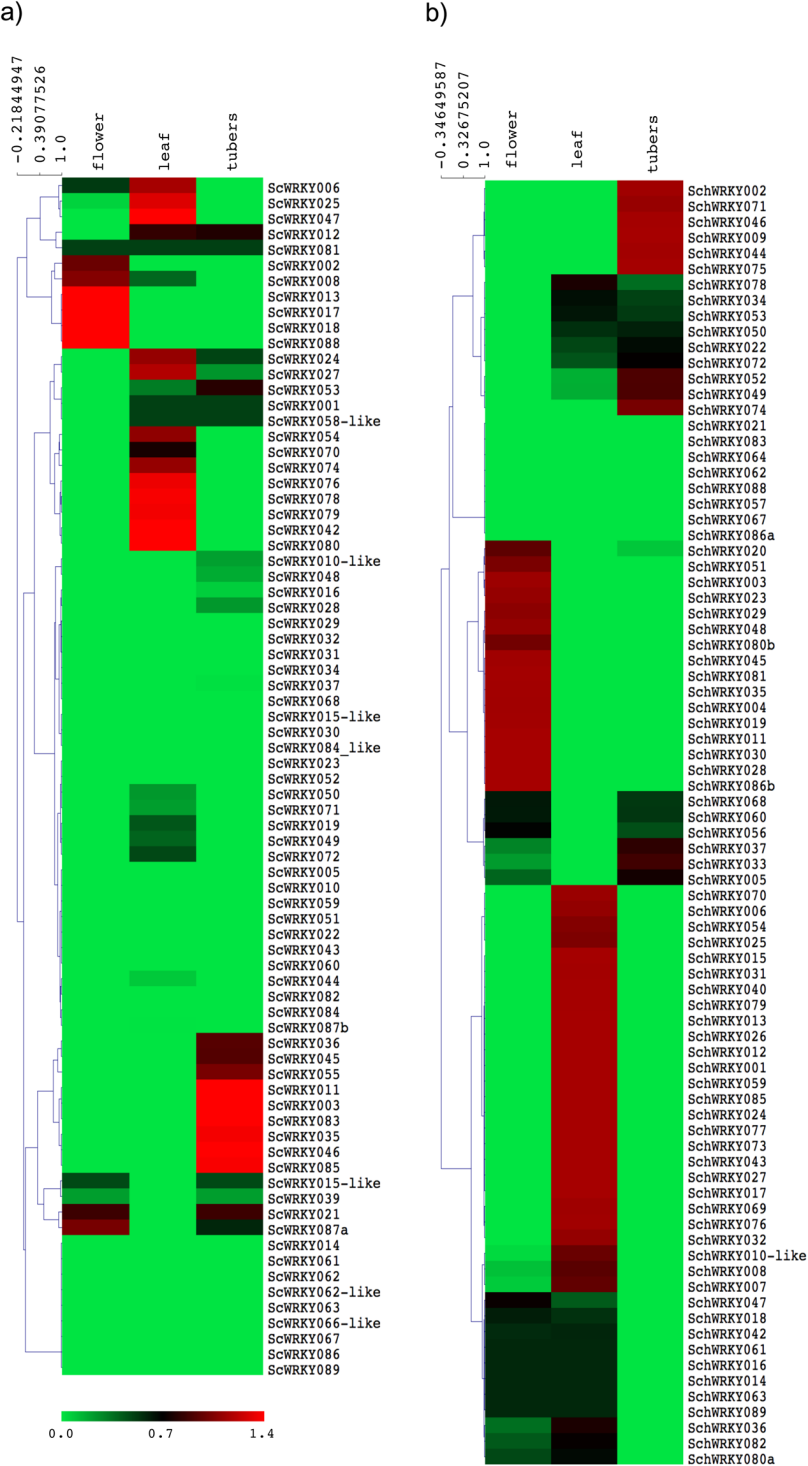
(**a**) Expression profile analysis of *ScWRKYs* genes in different tissues. Transcriptome data (Reads Per Kilobase per Million mapped reads; RPKM) were used to measure the expression levels of *ScWRKY* genes in leaves, tubers and flowers. The colored scale for the different expression levels is shown. b) Expression profile analysis of *SchWRKYs* genes in different tissues. Transcriptome data (Reads Per Kilobase per Million mapped reads; RPKM) were used to measure the expression levels of *SchWRKY* genes in leaves, tubers and flowers. The colored scale for the different expression levels is shown.

To explore the expression of *WRKY* genes, we analyzed and calculated the RNA sequence data available for leaf, flower and tuber in both species (Figs. 2a and 2b). The heat-map based expression profiles of *ScWRKYs* (Fig. 2a) and *SchWRKYs* (Fig. 2b) revealed their dynamic and differential expression in various tissues and that the range of expression varied among the two species. In *S. commersonii*, 21 (26.5%) *ScWRKY* genes (01, 14, 15-like, 15-like_2, 21, 30, 39, 58-like, 60, 61, 62, 62-like, 63, 66-like, 67, 81, 84_like, 85, 86, 88 and 89) showed very low or undetectable expression (FPKM values from 0 to 0.5) in all studied tissues, while 16 (20.2%) genes (18, 23, 03, 48, 87, 08, 11, 47, 79, 10, 51, 45, 05, 06, 12 and 49) were highly expressed (FPKM > 5) in all tissues. Some of the remaining genes showed tissue specificities. *ScWRKY002*, *ScWRKY013* and *ScWRKY017* were highly expressed only in flower, and *ScWRKY042* and *ScWRKY080* only in leaf, while no tuber specific *ScWRKYs* were identified. In *S. chacoense*, 21 (25%) *SchWRKY* genes (4, 14, 15, 16, 21, 34, 56, 57, 60, 61, 62, 63, 64, 67, 69, 81, 83, 86, 87a, 88 and 89) showed no expression in all considered tissues, while 42 (50%) were overexpressed in all tissues. Concerning the remaining genes, nine leaf-specific (*SchWRKY001, SchWRKY017, SchWRKY024, SchWRKY027, SchWRKY043, SchWRKY059, SchWRKY073, SchWRKY077* and *SchWRKY085)* and three flower-specific genes (*SchWRKY028, SchWRKY030* and *SchWRKY087b*) were identified. As is the case of *S. commersonii*, no tuber specific *SchWRKYs* were found.

**Figure 3:**
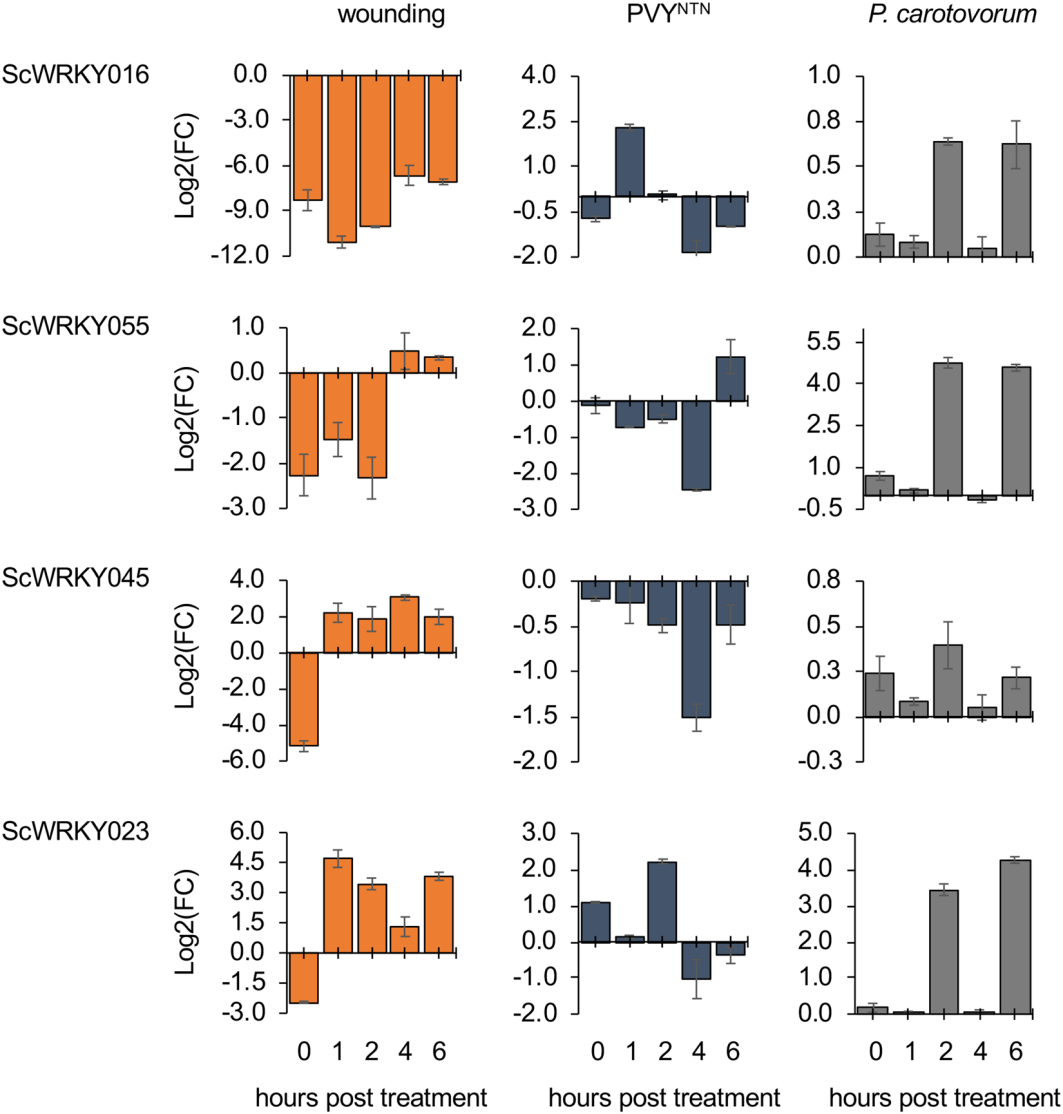
Expression RT-qPCR analysis of selected *ScWRKY* genes under abiotic and biotic stresses: wounding, PVY and *P. carotovorum*. For each stress the same time course of 0, 1, 2, 4, 6 hours post treatment was considered. The y-axes represent the mean relative expression normalized against non-treated plants for wounding stress and water-treated plants for PVY and *P. carotovorum* inoculations. Standard deviation values are shown.

Four *ScWRKY* genes (*ScWRKY016*, *ScWRKY023*, *ScWRKY045* and *ScWRKY055*) distributed in different Groups were selected to further investigate WRKYs behaviour in response to biotic (wounding) and abiotic (PVY and *P. carotovorum*) stressors using qRT-PCR (Fig. 3). The expression trend of our WRKYs was variable among and during treatments. In particular, wounding stress caused *ScWRKY023* and *ScWRKY045* overexpression during the whole treatment and *ScWRKY055* overexpression at 4- and 6-hours post treatment (hpt). As for viral infection response, *ScWRKY016* and *ScWRKY045* were always downregulated, while the other genes were upregulated only at one of the five hpt. The bacterial inoculation with *P. carotovorum* did not activate *ScWRKY016* and *ScWRKY045*, while the other two genes were upregulated at 2- and 6- hpt. Given the involvement of *WRKYs* in several biological processes, we wondered whether they might play roles under other stresses. Since WRKY expression data on wild potato species exposed to any stress are not available, we retrieved WRKYs RPKM values from *S. tuberosum* experiments involving several treatments and stressors. As shown in Figure S2, the transcription of most *WRKY* genes was affected by various treatments. Only *StWRKY61* to *StWRKY67* did not change their transcriptional activity upon stress. The late blight infection did not perturbate the expression of StWRKYs. *StWRKY023, StWRKY044, StWRKY054* and *StWRKY055* increased their expression following mannitol treatment, whereas ABA, IAA and GA3 hormonal treatments affected the transcriptional activity of 3 (*StWRKY027, StWRKY028* and *StWRKY046*), 1 (*StWRKY035*) and 4 (*StWRKY023, StWRKY054, StWRKY068, StWRKY070*) *S. tuberosum* WRKYs, respectively. BABA and BTH treatments induced an overexpression of 18 and 15 StWRKYs respectively, of which *StWRKY042, StWRKY075, StWRKY078* and *StWRKY080* were in common. Concerning heat stress, 12 *StWRKYs* were overexpressed. Finally, under *in-vitro* culture conditions, 10 *StWRKYs* were overexpressed in shoots and one (*StWRKY004)* in roots.

### In silico protein interaction network of selected ScWRKYs and SchWRKYs

A network of interaction was studied for WRKYs showing either tissue-specific or stress-induced expression (Figure S3a and S3b). The *S. commersonii* flower-specific expressed *WRKY002* formed a node with the anthocyanins and cell differentiation regulatory proteins. STRING analyses provided evidence that *ScWRKY002* interacts, among the others, with *JAF13* and *TTG1*, two well-characterized potato anthocyanins *bHLH* and *WD40* TFs^27,39^. Both the leaf-specific expressed *ScWRKY042* and *ScWRKY080* formed a cluster of interaction with a Leucine Rich Repeat (LRR) protein (an evolutionarily conserved protein associated with innate immunity in plants). The two wounding-responsive *ScWRKY023* and *ScWRKY045* established two independent nodes of interaction. The former set a cluster with “Wound-responsive Apetala2 like factor 2 (*WRAF2*)” (annotation for transcript PGSC0003DMT400021314 on SpudDB database), while *ScWRKY045* interacted with a cluster of proteins linked to a class of glycosyltransferase. Concerning *S. chacoense*, *SchWRKY030* (found to be flower-specific) interacted directly with *eIF2B*_5, a key protein involved in mRNA translation mechanisms. On the counterpart, the leaf-specific *SchWRKY017, SchWRKY043, SchWRKY059* and *SchWRKY077*, together with the flower-specific *SchWRKY028*, showed the same interaction with LRR proteins already described for *ScWRKY042* and *ScWRKY080*.

## Discussion

Due to its importance in the regulation of several processes in plants^5^, WRKY family has been studied in more than 60 plant species. In *Solanaceae*, data are available in some important crops, such as *S. tuberosum* (79^21^, 82^22^ and 81^12^ WRKYs), *S. lycopersicum* (83 WRKYs^41^) and *S. melongena* (50 WRKYs^42^). However, no information is available on the number and structural variability of WRKY TFs in *Solanaceae* wild species, which represent an important reservoir of genetic variation for breeding. This study was set up with the aim to profile WRKY encoding genes in *S. commersonii* and *S. chacoense*, two noteworthy tuber-bearing potato species used in potato breeding programs^20,43–45^.

### Structural analysis of ScWRKYs and SchWRKYs revealed interspecific diversification

The recently published genome annotation of *S. commersonii*^15^ and *S. chacoense*^16^ enables a comprehensive investigation of the WRKY family. We detected 79 and 84 genes encoding putative WRKY TFs in *S. commersonii* and *S. chacoense*, respectively. These results indicate that, compared to the cultivated potato^22^, *S. commersonii* possesses a lower number and *S. chacoense* a higher number of WRKY genes. Both species displayed a number of WRKYs greater than that of barley (45)^46^, castor bean (58)^47^, cucumber (55)^48^, rapeseed (43)^49^ and grapevine (59)^50^, and lower than that of cotton (120)^51^, maize (136)^52^, soybean (131)^53^ and rice (100)^25^. From this comparison, it appears that the number of WRKY encoding genes is not proportional to the genome size of the respective plant species, as also reported by Waqas *et al*.^54^. ScWRKY and SchWRKY proteins were primarily divided into three main phylogenetic Groups with Group II further classified into five subGroups (IIa-IIe). Most of WRKYs found in the two wild species belonged to Group II and this is in line with results obtained in *S. tuberosum*^22^. As known, WRKY proteins are characterized by one or more WRKY domain. In this study, we found that *ScWRKYs* and *SchWRKYs* had either one or two WDs. Interestingly, two *ScWRKYs* (*ScWRKY010* and *ScWRKY002)* carried three WDs. This might be the result of the acquisition of a WRKY domain during evolution, supporting findings of Aversano *et al*.^15^ and Esposito *et al*.^31,55^, who reported that *S. commersonii* prosper lineage-specific segmental duplications during evolution. Not only WDs number, but also WDs structural divergences identified in *S. commersonii* and *S. chacoense* might be the consequence of mutations during the process of evolution. Almost all *ScWRKYs* and *SchWRKYs* contained the highly conserved heptapeptide WRKYGQK motif, except for eight variants. Among them, WGKYGQK of *ScWRKY014*, WRWLKCG of *ScWRKY061*, WHKYGQK of *SchWRKY014* and WKKHGSN in *SchWRKY057* were not found in any other species. On the counterpart, the remaining variants were identified also in *S. tuberosum*^22^, *S. lycopersicum*^56^, *H. vulgare*^57^ and *C. annum*^58^. Zhou *et al.*^59^ hypothesized that these variations may change the DNA targets’ binding specificity. The structural diversity has been investigated also at the genomic level through the identification of exons and introns. As reported by Shiu and Bleecker^60^, this can highlight events of diversification and neo-functionalization of WRKY genes. In contrast to findings by Wang *et al*.^61^, our results did not reveal a conservation of gene structure among the members of the same Group, even though they allowed the discrimination of eight intron-lacking WRKYs (two *ScWRKYs* and six *SchWRKYs*). This is in agreement with results reported in the cultivated potato, where *StWRKY23* and *StWRKY24* had no introns^21^. Lynch *et al*.^62^ hypothesized that the intron turnover can be the result of reverse transcription of the mature mRNA followed by homologous recombination with intron-containing alleles.

### Identification of tissue-specific and stress responsive WRKYs in wild potatoes

WRKY TFs have been found to play important roles under abiotic stresses, such as drought^8^, heat^63^, wounding^50^, and biotic constraints, such as bacteria^59^ and viruses^64^. Tissue-specificity of WRKY genes has also been highlighted in different crops, such as pepper^58^, cotton^65^ and soybean^66^, elucidating their role in developmental and functional processes. Our study investigated for the first time the stress response and tissue-specificity of WRKY genes in two wild potato species. Six and 11 WRKY genes were identified as flower- and leaf-specific, respectively. Zhang and collaborators^21^ considered that the known protein-protein interaction network can provide important clues to better understand gene expression regulation. Basing on this, we investigated the interactome of tissue-specific and stress-responsive WRKYs identified here and found potential co-expression relationships between *ScWRKYs*/*SchWRKYs* and genes of various pathways. From our analyses, interesting observations and different clues for future functional studies have emerged. For example, *ScWRKY002* could be in some way involved in anthocyanin activation in flowers of *S. commersonii*: it interacts with anthocyanin bHLHs and the flower of this wild species strongly accumulates anthocyanins^14,67^. Previous studies reported that some WRKYs can be involved in the coordination of multiple biological processes. For example, *AtWRKY33* regulates disease resistance, NaCl tolerance and thermotolerance^68–70^, while *GhWRKY40* modulates tolerance to wounding stress and resistance to *R. solanacearum*^43^. This suggests that some WRKY proteins provide important nodes of crosstalk between different physiological processes. However, the putative members of WRKY family and their possible roles in signalling crosstalk are still barely known. To the authors’ best knowledge, no expression data are available on ScWRKYs and SchWRKYs; by contrast, StWRKYs have previously received attention. Among them, only Shahzad *et al*.^71^ and Yogendra *et al*.^72^ found StWRKY010 (PGSC0003DMP400029302) and StWRKY020 (PGSC0003DMP400028763) to be active in *P. infestans*-potato interaction. Consistently with these data, our results indicated that the same genes increased their expression after BABA treatment, known to confer protection against several biotic threats. Furthermore, we focused our attention on a group of proteins (ScWRKY016, ScWRKY023, ScWRKY045 and ScWRKY055) which were reported to be stress-responsive^21,73^. For these genes, we tested the transcriptional activity of wild *S. commersonii* alleles after wounding and bacterial infection and investigated on their direct orthologs expression following various treatments. Among them, StWRKY016, StWRKY045 and StWRKY055 appeared to be required by plants to face damages by heat stress, while StWRKY023 was reported to be active under mannitol and GA3 treatments as well as drought stress^73^. Our results suggested that the wild alleles of *ScWRKY023* and *ScWRKY045* might represent promising candidates for multiple stress responses as they are leaf-specific and constantly expressed after wounding in *S. commersonii* but not in the cultivated potato. In addition, WRKY023 is also induced by bacterial infection and it is suggested to interact with both a WRAF2-like protein and with the LRR mediated immunity system^74,75^.

## Conclusions

The present study identified 79 and 84 genes encoding putative WRKY TFs in *S. commersonii* and *S. chacoense*, respectively. Their protein structure and data from the comparative analyses suggested an interspecific variability of WRKY genes. Most of them were up-regulated under stress conditions and across different tissues, hinting a possible role in the cross-talk between plant and environmental cues in potato species. Taken as hole, these analyses will help to hasten the determination of the function of WRKY TFs especially in response to biotic and abiotic stresses. Candidate *ScWRKY* and *SchWRKY* genes identified here can be employed in potato breeding programs.

## Supplementary information

Supplementary information.
