# Supplementary material for: WRKY genes family study reveals tissue-specific and stress-responsive TFs in wild potato species
Source: Sci Rep. 2020 Apr 28;10:7196. doi: 10.1038/s41598-020-63823-w (PMC7188836; doi:10.1038/s41598-020-63823-w)
Supplement: Supplementary file 1 — Supplementary information. [file 41598_2020_63823_MOESM1_ESM.pdf]

WRKY genes family study reveals tissue-specific and stress-responsive TFs in wild potato species

Clizia Villano, Salvatore Esposito, Vincenzo D'Amelia, Raffaele Garramone, Daniela Alioto, Astolfo Zoina, Riccardo Aversano, Domenico Carputo

SUPPLEMENTARY INFORMATION

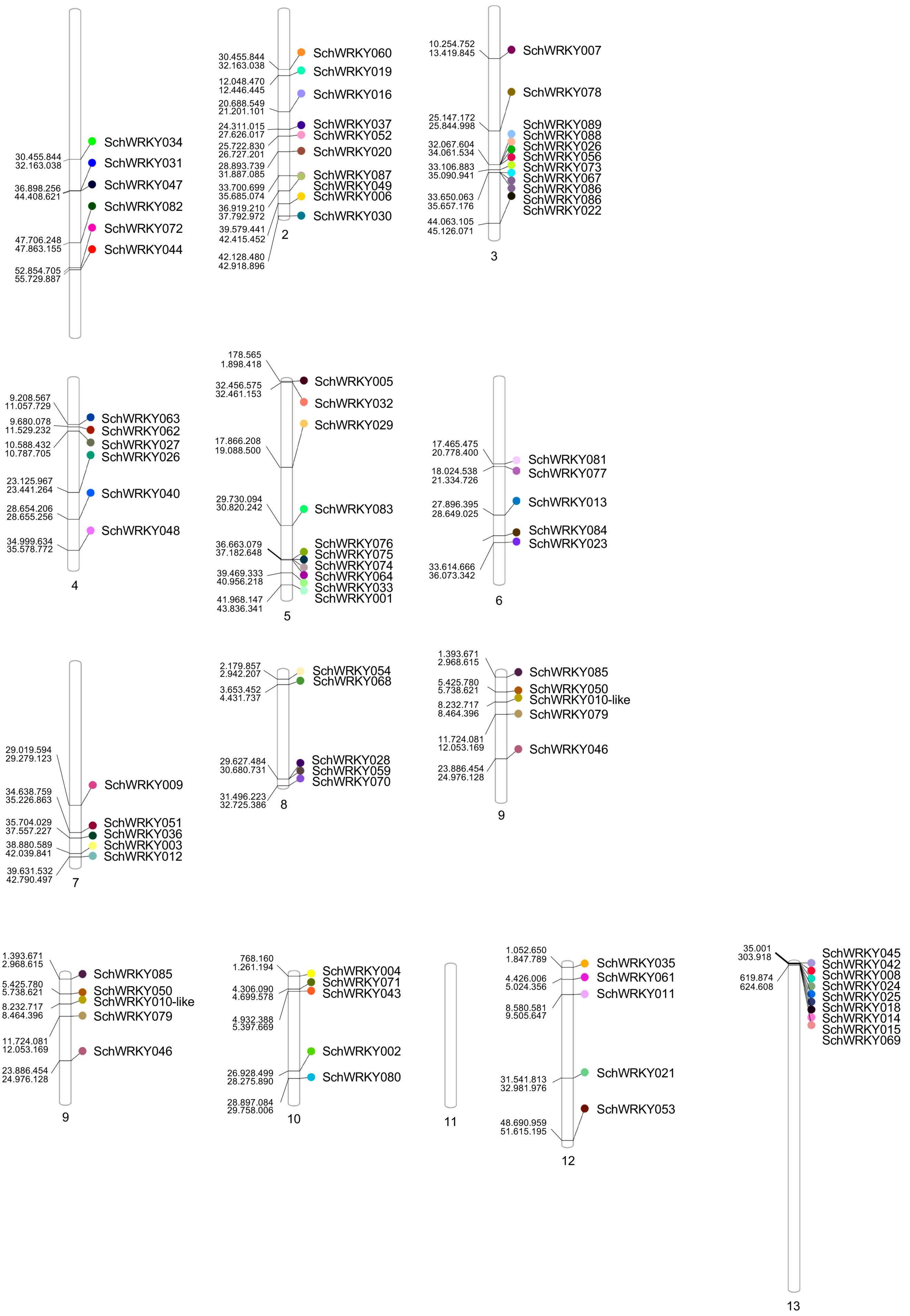

Figure S1. Linkage map of Group localization and distribution of WRKY genes in *S. chacoense*.

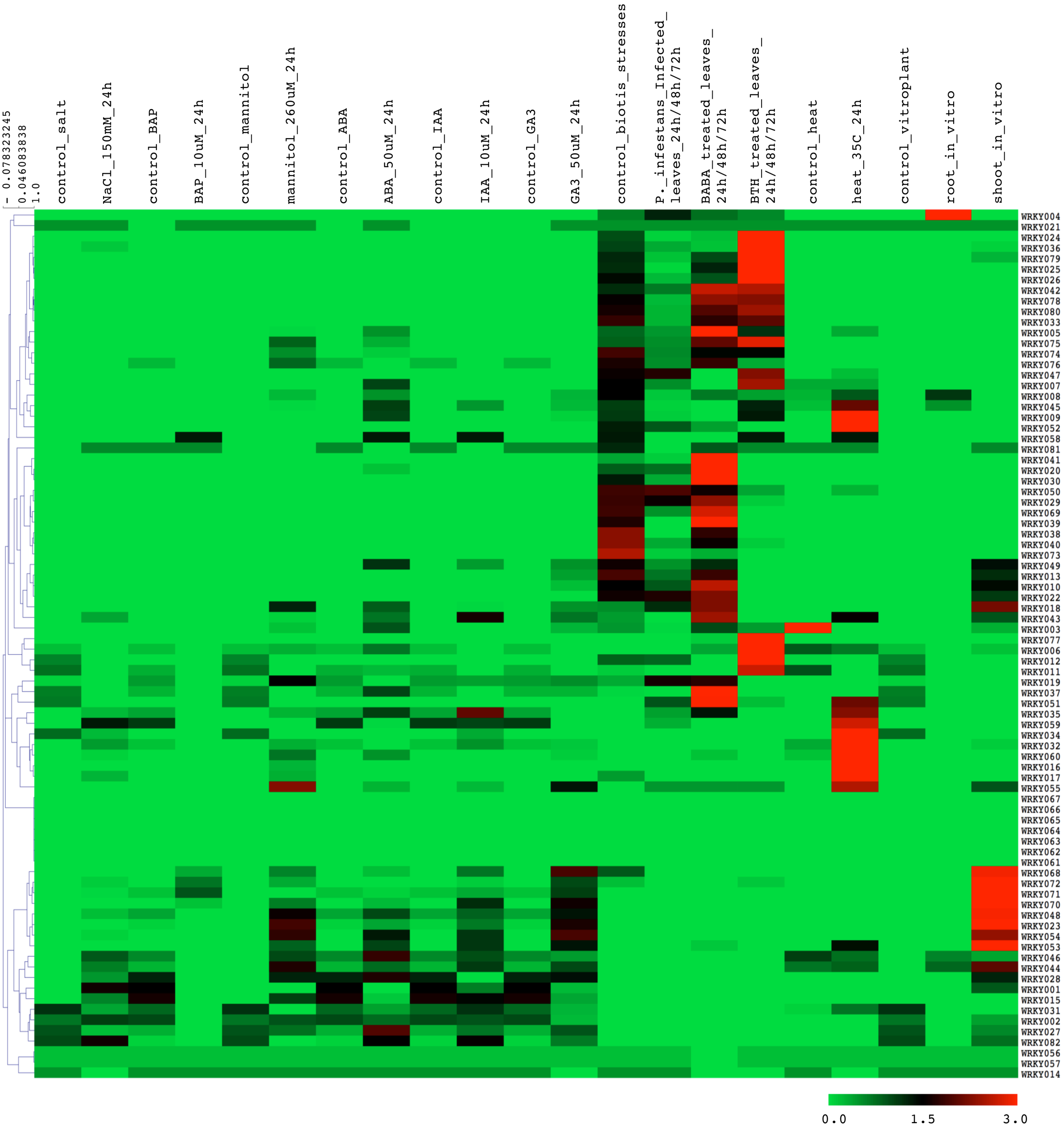

Figure S2. Expression profile analysis of *StWRKYs* genes following various treatments. Transcriptome data (Reads Per Kilobase per Million mapped reads; RPKM) were used to measure the expression levels of *StWRKY* genes in leaves after salt stress (50 mM NaCl for 24 h), osmotic stress (260 µM mannitol for 24 h), heat stress (35 °C for 24 h), treatments to abscisic acid (ABA) (50 µM for 24 h), indole-3-acetic acid (IAA) (10 µM for 24 h), gibberellic acid (GA3) (50 µM for 24 h), *P. infestans* (24, 48, 72 h), β-aminobutyric acid (BABA) (24, 48, 72 h), benzothiadiazole (BTH) (24, 48, 72 h), and following *in-vitro* growth (roots and shoots). The colored scale for the different expression levels is shown.

A

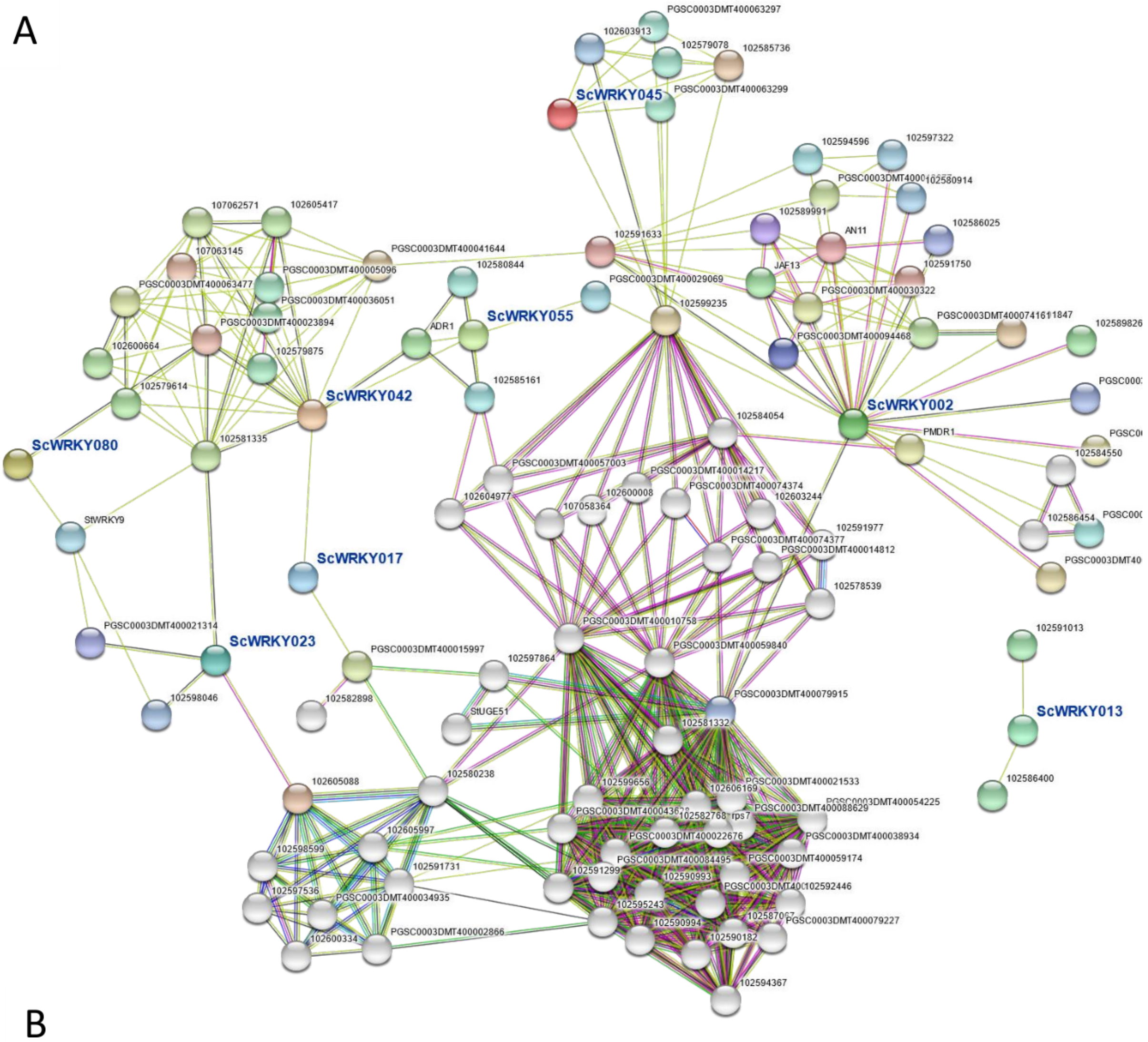

B

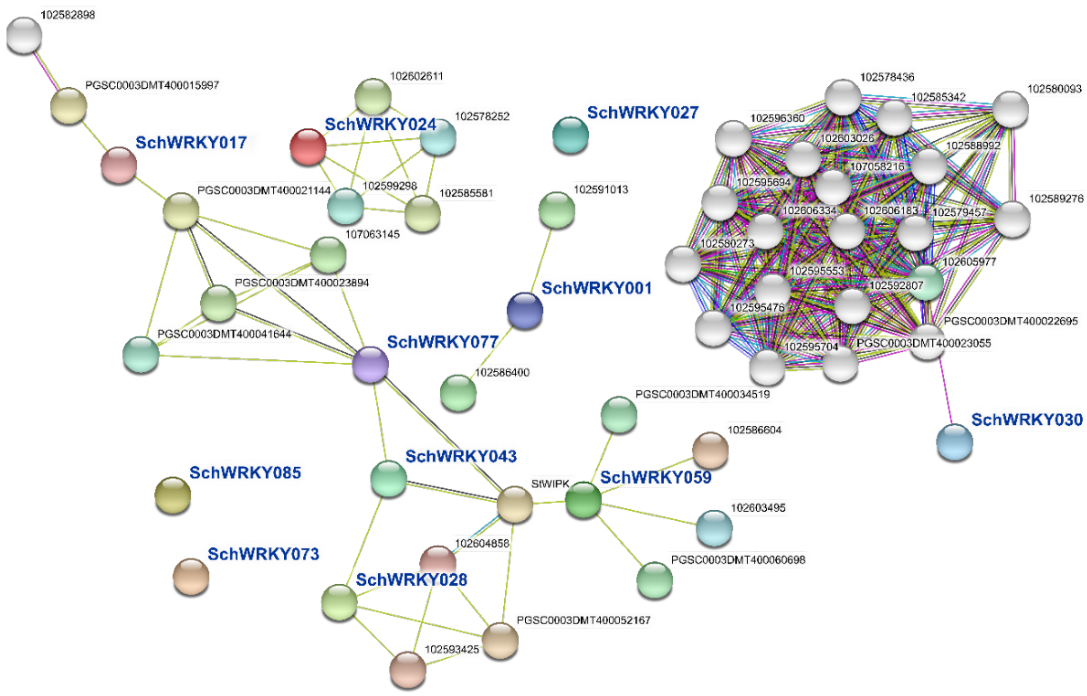

Figure S3. Protein-protein interaction of selected *WRKYs*.

Supplementary Table 1. List and sequence of primers used in the present study

Primer ID	Primer Sequence 5'-->3'
ScWRKY063_Fw	GCAAGTACGGTCAAAAGCAT
ScWRKY063_Rv	GTTGTGTTCACCGGAATAGG
ScWRKY016_Fw	GTTCGGACAAAATGCGATTA
ScWRKY016_Rv	CTTCGAGACATCGTTGGACT
ScWRKY045_Fw	GGCGGTTTCTACTTCAGCAG
ScWRKY045_Rv	TGGTCGGAGTACATGTCGAG
ScWRKY023_Fw	GCTCATTTGCACCTGGGT
ScWRKY023_Rv	TAATCGGCTGGCAGTGGA
ScWRKY035_Fw	GCCTTCATGACAAAGAGTGA
ScWRKY035_Rv	TGACCTTTCCACACGTTTC
ScWRKY055_Fw	TGAGGGTTCTTTATCTGGCG
ScWRKY055_Rv	TGGCACTGAAATTACTCTCTTC
StWRKY003_Fw	TTCAAGCCAGTTCCAGAGAC
StWRKY003_Rv	CGGTGAGATACAAGCGAGTT
StWRKY010_Fw	ACCTTCTTCCATCTCCGAC
StWRKY010_Rv	CAGCTGTCCCTACTTGAG
StWRKY044_Fw	GGACAAAAACCCATCAAAGG
StWRKY044_Rv	GAGGGAATTCCGGTGAGTA
StWRKY080_Fw	GTTGCAAGATTTCGTCATTGA
StWRKY080_Rv	TGGCCATCATACACCAAAGT
StWRKY079_Fw	GTCAAGTCGCCGGAAGAT
StWRKY079_Rv	CACCTAAAGTAGTGCCTTGGA
StWRKY049_Fw	GGCTGAAGAGGGTAGTTCGA
StWRKY049_Rv	TGTTTACGTGCTGGACAACC

Supplementary Table 2. Structural characteristics of *ScWRKYs* and *SchWRKYs* including protein length, conserved heptapeptide, zinc-finger type and number of WRKY domains, exons and introns.

WRKYs	Locus ID	Scaffold/chromosome	Start	End	Strand	Protein lenght	Conserved heptapeptide	Zinc-finger type	# WD	# exon	# intron
ScWRKY001	maker_scaffold1882_snap_gene_0_38_mRNA_1	scaffold1882	8259	11118	+	481	WRKYGQK/WRKYGQK	C-X4-C-X22-H-X-H/C-X4-C-X23-H-X-H	2	3	2
ScWRKY002	maker_scaffold7854_augustus_gene_0_54_mRNA_1	scaffold7854	60515	63920	+	537	WRKYGQK/WRKYGQK/WRKYGQK	C/C-X4-C-X22-H-X-H/C-X4-C-X23-H-X-H	3	5	4
ScWRKY003	maker_scaffold2503_augustus_gene_0_43_mRNA_1	scaffold2503	22712	28376	-	743	WRKYGQK/WRKYGQK	C-X4-C-X22-H-X-H/C-X4-C-X23-H-X-H	2	6	5
ScWRKY005	maker_scaffold31249_augustus_gene_0_94_mRNA_1	scaffold31249	59454	65169	-	508	WRKYGQK/WRKYGQK	C-X4-C-X22-H-X-H/C-X4-C-X23-H-X-H	2	4	3
ScWRKY006	augustus_masked_scaffold354_abinit_gene_0_10_mRNA_1	scaffold354	49718	52502	-	458	WRKYGQK/WRKYGQK	C-X4-C-X22-H-X-H/C-X4-C-X23-H-X-H	2	4	3
ScWRKY008	maker_scaffold9215_augustus_gene_0_73_mRNA_1	scaffold9215	42740	45983	-	559	WRKYGQK/WRKYGQK	C-X4-C-X22-H-X-H/C-X4-C-X23-H-X-H	2	4	3
ScWRKY010	maker_scaffold440_augustus_gene_0_51_mRNA_1	scaffold440	12332	15746	+	660	WRKYGQK/WRKYGQK/WRKYGQK	C-X4-C-X22-H-X-H/C-X4-C-X22-H-X-H/C-X4-C-X23-H-X-H	3	6	5
ScWRKY010-like	maker_scaffold5761_snap_gene_0_26_mRNA_1	scaffold5761	17673	20769	+	528	WRKYGQK/WRKYGQK	C-X4-C-X22-H-X-H/C-X4-C-X23-H-X-H	2	5	4
ScWRKY011	maker_scaffold1729_augustus_gene_0_61_mRNA_1	scaffold1729	58064	64968	-	611	WRKYGQK/WRKYGQK	C-X4-C-X22-H-X-H/C-X4-C-X23-H-X-H	2	6	5
ScWRKY012	genemark_scaffold41213_abinit_gene_0_8_mRNA_1	scaffold41213	16070	21785	-	735	WRKYGQK/WRKYGQK	C-X4-C-X22-H-X-H/C-X4-C-X23-H-X-H	2	7	6
ScWRKY013	genemark_scaffold21247_abinit_gene_0_14_mRNA_1	scaffold21247	5578	6186	+	225	WRKYGQK	C-X4-C-X22-H-X-H	1	1	0
ScWRKY014	augustus_masked_scaffold89_abinit_gene_0_4_mRNA_1	scaffold89	61770	65094	+	145	WGKYGQK	C-X4-C-X22-H-X-H	1	4	3
ScWRKY015-like	augustus_masked_scaffold10618_abinit_gene_0_2_mRNA_1	scaffold10618	28276	34545	-	664	WRKYGQK	C-X5-C-X23-H-X-H	1	8	7
ScWRKY015-like_2	maker_scaffold5837_augustus_gene_0_23_mRNA_1	scaffold5837	35374	37850	-	369	WRKYGQK/WRKYGQK	C-X5-C/C-X5-C-X23-H-X-H	2	4	3
ScWRKY016	maker_scaffold13177_augustus_gene_0_11_mRNA_1	scaffold13177	6097	8406	+	446	WRKYGQK	C-X5-C-X23-H-X-H	1	3	2
ScWRKY017	maker_scaffold17033_augustus_gene_1_25_mRNA_1	scaffold17033	103259	105449	-	480	WRKYGQK	C-X5-C-X23-H-X-H	1	5	4
ScWRKY018	maker_scaffold24758_augustus_gene_0_43_mRNA_1	scaffold24758	39833	42648	-	660	WRKYGQK	C-X5-C-X23-H-X-H	1	5	4
ScWRKY019	maker_scaffold11314_augustus_gene_0_23_mRNA_1	scaffold11314	6921	9167	-	494	WRKYGQK	C-X5-C-X23-H-X-H	1	5	4
ScWRKY021	maker_scaffold31159_snap_gene_0_73_mRNA_1	scaffold31159	33273	34286	-	189	WRKYGQK	C-X5-C-X23-H-X-H	1	3	2
ScWRKY022	maker_scaffold2968_augustus_gene_0_60_mRNA_1	scaffold2968	69485	72344	+	488	WRKYGQK/WRKYGQK	none/C-X5-C-X23-H-X-H	2	7	6
ScWRKY023	maker_scaffold9305_augustus_gene_0_17_mRNA_1	scaffold9305	3578	5333	+	366	WRKYGQK	C-X5-C-X23-H-X-H	1	4	3
ScWRKY024	maker_scaffold27786_augustus_gene_0_38_mRNA_1	scaffold27786	49221	50932	-	235	WRKYGQK	C-X5-C-X23-H-X-H	1	3	2
ScWRKY025	maker_scaffold38372_augustus_gene_0_21_mRNA_1	scaffold38372	15016	17000	-	253	WRKYGQK	C-X5-C-X23-H-X-H	1	4	3
ScWRKY027	augustus_masked_scaffold4984_abinit_gene_0_0_mRNA_1	scaffold4984	15058	23355	+	317	WRKYGQK/WRKYGQK	none/C-X4-C-X23-H-X-H	2	4	3
ScWRKY028	augustus_masked_scaffold1174_abinit_gene_0_6_mRNA_1	scaffold1174	55115	57163	-	228	WRKYGQK	C-X4-C-X23-H-X-H	1	2	1
ScWRKY029	maker_scaffold7139_snap_gene_1_53_mRNA_1	scaffold7139	172010	174504	-	172	WRKYGQK	C-X4-C-X23-H-X-H	1	2	1
ScWRKY030	augustus_masked_scaffold7796_abinit_gene_0_0_mRNA_1	scaffold7796	17391	19402	+	142	WRKYGQK	C-X4-C-X23-H-X-H	1	2	1
ScWRKY031	maker_scaffold1339_augustus_gene_0_64_mRNA_1	scaffold1339	59575	62095	+	316	WRKYGQK	C-X4-C-X23-H-X-H	1	3	2
ScWRKY032	maker_scaffold8864_snap_gene_1_45_mRNA_1	scaffold8864	121955	130271	+	328	WRKYGQK	C-X4-C-X23-H-X-H	1	4	3
ScWRKY034	genemark_scaffold11173_abinit_gene_0_31_mRNA_1	scaffold11173	29149	30734	-	322	WRKYGQK	C-X4-C-X23-H-X-H	1	3	2
ScWRKY035	maker_scaffold23185_snap_gene_0_11_mRNA_1	scaffold23185	983	2868	-	171	WRKYGQK	C-X4-C-X23-H-X-H	1	2	1
ScWRKY036	maker_scaffold7687_snap_gene_0_74_mRNA_1	scaffold7687	71842	79645	+	576	WRKYGQK/WRKYGQK	C-X7-H-X27-H-X-H/C-X4-C-X23-H-X-H	2	6	5
ScWRKY037	maker_scaffold230_augustus_gene_0_11_mRNA_1	scaffold230	20902	22612	-	318	WRKYGQK	C-X4-C-X22-H-X-H	1	3	2
ScWRKY039	maker_scaffold24623_snap_gene_0_85_mRNA_1	scaffold24623	81485	83890	-	238	WRKYGKK	C-X4-C-X23-H-X-H	1	3	2
ScWRKY042	maker_scaffold3388_augustus_gene_0_57_mRNA_1	scaffold3388	53316	56538	+	184	WRKYGQK	C-X4-C-X23-H-X-H	1	3	2
ScWRKY043	maker_scaffold3609_augustus_gene_0_44_mRNA_1	scaffold3609	28204	30279	+	298	none	C-X5-C-X23-H-X-H	1	2	1
ScWRKY044	maker_scaffold9525_snap_gene_0_21_mRNA_1	scaffold9525	5183	9570	-	736	WRKYGQK/WRKYGQK	C-X5-C-X23-H-X-H/C-X5-C-X23-H-X-H	2	7	6
ScWRKY045	maker_scaffold3826_snap_gene_0_9_mRNA_1	scaffold3826	13511	16538	-	318	WRKYGQK	C-X5-C-X23-H-X-H	1	5	4
ScWRKY046	maker_scaffold36167_augustus_gene_0_25_mRNA_1	scaffold36167	15196	17537	+	346	WRKYGQK	C-X5-C-X23-H-X-H	1	3	2
ScWRKY047	maker_scaffold13022_augustus_gene_0_10_mRNA_1	scaffold13022	9673	11971	-	410	WRKYGQK/WRKYGQK	none/C-X5-C-X23-H-X-H	2	6	5
ScWRKY048	maker_scaffold13210_augustus_gene_0_60_mRNA_1	scaffold13210	81088	82775	+	354	WRKYGQK	C-X5-C-X23-H-X-H	1	3	2
ScWRKY049	maker_scaffold20941_augustus_gene_0_53_mRNA_1	scaffold20941	23044	24244	+	324	WRKYGQK	C-X5-C-X23-H-X-H	1	3	2
ScWRKY050	maker_scaffold37089_augustus_gene_0_11_mRNA_1	scaffold37089	3661	5682	+	330	WRKYGQK	C-X5-C-X23-H-X-H	1	3	2
ScWRKY051	maker_scaffold23049_snap_gene_0_12_mRNA_1	scaffold23049	197	3330	+	229	WRKYGQK	C-X5-C-X23-H-X-H	1	2	1
ScWRKY052	maker_scaffold10802_augustus_gene_0_67_mRNA_1	scaffold10802	12303	14161	-	300	WRKYGQK	C-X5-C-X23-H-X-H	1	3	2
ScWRKY053	maker_scaffold27282_augustus_gene_1_73_mRNA_1	scaffold27282	166391	167947	-	313	WRKYGQK	C-X5-C-X23-H-X-H	1	3	2
ScWRKY054	maker_scaffold16944_snap_gene_0_17_mRNA_1	scaffold16944	42	1477	+	337	WRKYGQK/WRKYGQK	C-X5-C-X22-H-X-H/C-X5-C-X23-H-X-H	2	6	5
ScWRKY055	maker_scaffold15104_snap_gene_0_20_mRNA_1	scaffold15104	11864	16690	-	261	WRKYGQK	C-X5-C-X23-H-X-H	1	3	2
ScWRKY058-like	maker_scaffold19913_augustus_gene_0_9_mRNA_1	scaffold19913	4898	6675	-	276	WRKCGQK	C-X5-C-X23-H-X-H	2	4	3
ScWRKY059	maker_scaffold1174_augustus_gene_0_58_mRNA_1	scaffold1174	68724	70826	-	310	WRKYGQK	C-X5-C-X23-H-X-H	1	3	2
ScWRKY060	augustus_masked_scaffold18408_abinit_gene_0_2_mRNA_1	scaffold18408	14208	17079	-	411	WRKYGQK	C-X5-C-X23-H-X-H	1	3	2
ScWRKY061	genemark_scaffold32401_abinit_gene_0_3_mRNA_1	scaffold32401	11612	12289	+	200	WRWLKCG	none	1	2	1
ScWRKY062	augustus_masked_scaffold5103_abinit_gene_0_3_mRNA_1	scaffold5103	20889	22081	-	219	WSKYGQK	C-X5-C-X25-H	1	4	3
ScWRKY062-like	maker_scaffold1081_snap_gene_0_34_mRNA_1	scaffold1081	68071	70685	+	310	none	none	1	4	3
ScWRKY063	maker_scaffold1081_snap_gene_0_35_mRNA_1	scaffold1081	30405	32843	-	341	WRKYGQK	C-X5-C-X23-H-X-H	1	4	3
ScWRKY064	maker_scaffold17826_snap_gene_0_36_mRNA_1_like	scaffold17826	4879	6337	-	363	WRKYGQK	C-X4-H-X24-H-X-H	1	5	4
ScWRKY065	genemark_scaffold17826_abinit_gene_0_25_mRNA_1_like	scaffold17826	48569	49788	-	310	WRKYGQK	none	1	3	2
ScWRKY066-like	maker_scaffold35381_snap_gene_0_8_mRNA_1	scaffold35381	1835	3442	+	373	WRKCGQK	C-X5-C-X23-H-X-H	1	4	3
ScWRKY067	maker_scaffold1552_snap_gene_0_60_mRNA_1	scaffold1552	11943	23856	+	738	WRKYGQK	C-X5-C-X23-H-X-H	1	7	6
ScWRKY068	genemark_scaffold25887_abinit_gene_0_18_mRNA_1	scaffold25887	17512	19679	+	217	WRKYGQK	C-X8-C-X27-H-X2-H	1	3	2
ScWRKY070	maker_scaffold30616_augustus_gene_0_60_mRNA_1	scaffold30616	28647	30485	-	339	WRKYGQK	C-X7-C-X23-H-X-C	1	3	2
ScWRKY071	maker_scaffold12441_snap_gene_0_28_mRNA_1	scaffold12441	22505	23812	-	271	WRKYGQK	C-X7-C-X23-H-X-C	1	3	2
ScWRKY072	maker_scaffold12583_augustus_gene_0_32_mRNA_1	scaffold12583	6422	9021	+	344	WRKYGQK	C-X7-C-X23-H-X-C	1	3	2
ScWRKY074	maker_scaffold31861_augustus_gene_0_50_mRNA_1	scaffold31861	68357	71876	+	272	WRKYGQK/WRKYGQK	C-X1-C-X26-H-X-C/C-X4-C-X23-H-X-C	2	3	2
ScWRKY076	snap_masked_scaffold31861_abinit_gene_0_36_mRNA_1	scaffold31861	39042	40241	-	243	WRKYGQK	C-X4-C-X23-H-X-C	1	2	1
ScWRKY078	maker_scaffold978_augustus_gene_1_25_mRNA_1	scaffold978	139276	141436	+	293	WRKYGQK	C-X4-C-X23-H-X-C	2	2	1
ScWRKY079	maker_scaffold3600_augustus_gene_0_37_mRNA_1	scaffold3600	8178	9937	+	216	WRKYGQK	C-X7-C-X23-H-X-C	1	3	2

WRKYs	Locus ID	Scaffold/chromosome	Start	End	Strand	Protein lenght	Conserved heptapeptide	Zinc-finger type	# WD	# exon	# intron
ScWRKY080	maker_scaffold15162_snap_gene_0_46_mRNA_1	scaffold15162	3	2198	-	387	WRKYGQK	C-X7-C-X23-H-X-C	1	3	2
ScWRKY081	maker_scaffold7208_snap_gene_0_38_mRNA_1	scaffold7208	11527	17366	-	193	WRKY	none	1	5	4
ScWRKY082	augustus_masked_scaffold568_abinit_gene_0_2_mRNA_1	scaffold568	31816	33523	+	235	WRKYGQK	none	1	3	2
ScWRKY083	augustus_masked_scaffold6878_abinit_gene_0_0_mRNA_1	scaffold6878	4081	5121	+	404	none	C-X5-C-X23-H-X-H	1	2	1
ScWRKY084	augustus_masked_scaffold10960_abinit_gene_0_3_mRNA_1	scaffold10960	34833	38181	+	628	WRKYGQK	C-X5-C-X23-H-X-H	1	4	3
ScWRKY084_like	maker_scaffold5413_augustus_gene_0_50_mRNA_1	scaffold5413	10659	15440	+	303	WRKYGQK	C-X5-C-X23-H-X-H	1	6	5
ScWRKY085	augustus_masked_scaffold12000_abinit_gene_0_1_mRNA_1	scaffold12000	12631	14378	-	196	WRKYGQK	C-X4-C-X23-H-X-H	1	3	2
ScWRKY087a	maker_scaffold2503_augustus_gene_0_39_mRNA_1	scaffold2503	14091	21915	+	752	WRKYGQK	C-X4-C-X23-H-X-H	1	5	4
ScWRKY087b	snap_masked_scaffold25887_abinit_gene_0_23_mRNA_1	scaffold25887	768	1280	+	161	WRKYGQK	C-X4-C-X23-H-X-H	1	1	0
ScWRKY088	maker_scaffold12465_snap_gene_0_41_mRNA_1	scaffold12465	21252	35573	+	1385	WRKYGMK	C-X5-C-X23-H-X-H	1	8	7
ScWRKY089	maker_scaffold42117_snap_gene_0_27_mRNA_1	scaffold42117	68009	71036	-	106	WRKYGQK	C-X4-C-X23-H-X-H	1	2	1
SchWRKY001	g8177.t1	Chr05	41968147	43836341	-	482	WRKYGQK/WRKYGQK	C-X5-C-X22-H-X-H/C-X5C-X23-H-X-H	2	5	4
SchWRKY002	g13037.t1	Chr10	26928499	28275890	-	468	WRKYGQK/WRKYGQK	C-X5-C-X22-H-X-H/C-X4-C-X23-H-X-H	2	7	6
SchWRKY003	g1652.t1	Chr07	38880589	42039841	+	744	WRKYGQK/WRKYGQK	C-X5-C-X22-H-X-H/C-X4-C-X23-H-X-H	2	7	6
SchWRKY004	g27614.t1	Chr10	768160	1261194	+	741	WIKYGEN/WRKYGQK	C-X4-C-X22-H-X-H/C-X4-C-X23-H-X-H	2	6	5
SchWRKY005	g9868.t1	Chr05	178565	1898418	-	508	WRKYGQK/WRKYGQK	C-X5-C-X22-H-X-H/C-X4-C-X23-H-X-H	2	4	3
SchWRKY006	g2882.t1	Chr02	39579441	42415452	+	459	WRKYGQK/WRKYGQK	C-X5-C-X22-H-X-H/C-X4-C-X23-H-X-H	2	4	3
SchWRKY007	g5502.t1	Chr03	10254752	13419845	-	517	WRKYGQK/WRKYGQK	C-X5-C-X22-H-X-H/C-X4-C-X23-H-X-H	2	4	3
SchWRKY008	g35580.t1	ChrUn	75562	82373	+	550	WRKYGQK/WRKYGQK	C-X5-C-X22-H-X-H/C-X5-C-X23-H-X-H	2	6	5
SchWRKY009	g34576.t1	Chr07	29019594	29279123	-	334	WRKYGQK/WRKYGQK	C-X4-C-X22-H-X-H/C-X4-C-X23-H-X-H	2	5	4
SchWRKY010-like	g35137.t1	Chr09	8232717	8464396	-	526	WRKYGQK/WRKYGQK	C-X4-C-X22-H-X-H/C-X4-C-X22-H-X-H	2	5	4
SchWRKY011	g18246.t1	Chr12	8580581	9505647	-	608	WRKYGQK/WRKYGQK	C-X5-C-X22-H-X-H/C-X4-C-X23-H-X-H	2	6	5
SchWRKY012	g1746.t1	Chr07	39631532	42790497	-	599	WRKYGQK/WRKYGQK	C-X5-C-X22-H-X-H/C-X4-C-X23-H-X-H	2	6	5
SchWRKY013	g22208.t1	Chr06	27896395	28649025	-	398	WRKYGQK/WRKYGQK	C-X5-C-X22-H-X-H/none	2	4	3
SchWRKY014	g31999.t1	ChrUn	303433	303918	+	162	WHKYGQK	C-X5-C-X22-H-X-H	1	1	0
SchWRKY015	g31090.t1	ChrUn	346835	350835	-	521	WRKYGQK	C-X5-C-X23-H-X-H	1	5	4
SchWRKY016	g27534.t1	Chr02	20688549	21201101	+	414	WRKYGQK	C-X5-C-X23-H-X-H	1	4	3
SchWRKY017	g9538.t1	Chr01	58651449	60432788	-	478	WRKYGQK	C-X5-C-X23-H-X-H	1	5	4
SchWRKY018	g30842.t1	ChrUn	262883	265898	-	612	WRKYGQK	C-X6-C-X23-H-X-H	1	5	4
SchWRKY019	g8360.t1	Chr02	13305424	15262631	-	502	WRKYGQK	C-X5-C-X23-H-X-H	1	5	4
SchWRKY020	g2454.t1	Chr02	28893739	31887085	-	552	WRKYGQK	C-X5-C-X23-H-X-H	1	6	5
SchWRKY021	g11533.t1	Chr12	31541813	32981976	-	123	WRKYGQK	C-X5-C-X23-H-X-H	1	2	1
SchWRKY022	g16975.t1	Chr03	44063105	45126071	+	359	WRKYGQK	C-X4-C-X23-H-X-H	1	5	4
SchWRKY023	g5351.t1	Chr06	33614666	36073342	-	368	WRKYGQK	C-X5-C-X23-H-X-H	1	4	3
SchWRKY024	g31307.t1	ChrUn	232304	234313	-	262	WRKYGQK	C-X5-C-X23-H-X-H	1	4	3
SchWRKY025	g31308.t1	ChrUn	250556	252955	-	254	WRKYGQK	C-X5-C-X23-H-X-H	1	4	3
SchWRKY026	g32923.t1	Chr04	23125967	23441264	-	175	WRKYGQK	C-X4-C-X23-H-X-H	1	3	2
SchWRKY027	g36153.t1	Chr04	10588432	10787705	-	238	WRKYGQK	C-X4-C-X23-H-X-H	1	3	2
SchWRKY028	g15762.t1	Chr08	29627484	30666711	-	172	WRKYGQK	none	1	1	0
SchWRKY029	g14398.t1	Chr05	17866208	19088500	-	173	WRKYGQK	C-X4-C-X23-H-X-H	1	2	1
SchWRKY030	g20531.t1	Chr02	42128480	42918896	+	142	WRKYGQK	C-X5-C-X23-H-X-H	1	2	1
SchWRKY031	g5592.t1	Chr01	36898256	44286566	+	322	WRKYGQK	C-X4-C-X23-H-X-H	1	3	2
SchWRKY032	9895.t1	Chr10	32456575	32461153	-	323	WRKYGQK	C-X4-C-X23-H-X-H	1	9	8
SchWRKY033	g11063.t1	Chr05	39469333	40956218	+	268	WRKYGQK	C-X4-C-X23-H-X-H	1	3	2
SchWRKY034	g10072.t1	Chr01	30455844	32163038	-	337	WRKYGQK	C-X4-C-X23-H-X-H	1	3	2
SchWRKY035	g20677.t1	Chr12	1052650	1847789	-	336	WRKYGQK	C-X4-C-X23-H-X-H	1	3	2
SchWRKY036	g9069.t1	Chr07	35704029	37557227	-	320	WRKYGQK	C-X4-C-X23-H-X-H	1	3	2
SchWRKY037	g1078.t1	Chr04	24311015	27626017	-	319	WRKYGQK	C-X4-C-X23-H-X-H	1	3	2
SchWRKY040	g17334.t1	Chr02	28654206	28655256	-	172	WRKYGQK	none	1	2	1
SchWRKY042	g39518.t1	ChrUn	41795	43401	+	171	WRKYGKK	C-X4-C-X23-H-X-H	1	3	2
SchWRKY043	g28733.t1	Chr10	4932388	5397669	-	322	WRKYGQK	C-X5-C-X23-H-X-H	1	3	2
SchWRKY044	g4268.t1	Chr01	53228093	55729887	-	352	WRKYGQK	C-X5-C-X23-H-X-H	1	3	2
SchWRKY045	g37677.t1	ChrUn	35001	37721	+	350	WRKYGQK	C-X5-C-X23-H-X-H	1	3	2
SchWRKY046	g15556.t1	Chr09	23886454	24976128	-	347	WRKYGQK	C-X4-C-X23-H-X-H	1	3	2
SchWRKY047	g5604.t1	Chr01	37020941	44408621	-	245	WRKYGQK	C-X5-C-X23-H-X-H	1	3	2
SchWRKY048	g25995.t1	Chr04	34999634	35578772	-	353	WRKYGQK	C-X5-C-X23-H-X-H	1	3	2
SchWRKY049	g19483.t1	Chr02	36919210	37792972	-	325	WRKYGQK	C-X5-C-X23-H-X-H	1	3	2
SchWRKY050	g33267.t1	Chr09	5425780	5738621	+	325	WRKYGQK	C-X5-C-X23-H-X-H	1	3	2
SchWRKY051	g25365.t1	Chr07	34638759	35226863	+	280	WRKYGQK	C-X5-C-X23-H-X-H	1	3	2
SchWRKY052	g17075.t1	Chr02	25722830	26727201	+	302	WRKYGQK	C-X5-C-X23-H-X-H	1	3	2
SchWRKY053	g3467.t1	Chr12	48690959	51615195	+	318	WRKYGQK	C-X5-C-X23-H-X-H	1	3	2
SchWRKY054	g22375.t1	Chr08	2179857	2942207	+	335	WRKYGQK	C-X5-C-X23-H-X-H	1	3	2
SchWRKY056	g9378.t1	Chr03	32077977	34061534	-	261	WRKYGMK	C-X5-C-X23-H-X-H	1	1	0
SchWRKY057	g9376.t1	Chr03	32074058	34057408	-	192	WKKHGSN	C-X6-C-X23-H-X-H	1	1	0
SchWRKY059	g15764.t1	Chr08	29639951	30680731	-	311	WRKYGQK	C-X5-C-X23-H-X-H	1	3	2
SchWRKY060	g30025.t1	Chr02	12048470	12446445	-	412	WRKYGQK	C-X5-C-X23-H-X-H	1	3	2

WRKYs	Locus ID	Scaffold/chromosome	Start	End	Strand	Protein lenght	Conserved heptapeptide	Zinc-finger type	# WD	# exon	# intron
SchWRKY061	g24860.t1	Chr12	4426006	5024356	-	338	WHKCGQK	C-X5-C-X23-H-X-H	1	3	2
SchWRKY062	g9225.t1	Chr04	9680078	11529232	-	207	none	C-X5-C-X23-H-X-H	1	2	1
SchWRKY063	g9218.t1	Chr04	9208567	11057729	-	323	WRKYGQK	C-X5-C-X23-H-X-H	1	3	2
SchWRKY064	g32306.t1	Chr05	36861256	37182648	-	331	WRKYGQK	C-X6-C-X23-H-X-H	1	3	2
SchWRKY067	g9511.t1	Chr03	33650063	33651056	-	212	WRKYGQK	none	1	3	2
SchWRKY068	g21153.t1	Chr08	3653452	4431737	+	361	WRKYGQK	C-X7-C-X23-H-X-C	1	3	2
SchWRKY069	g24755.t1	ChrUn	619874	624608	+	353	WRKYGQK	C-X7-C-X23-H-X-C	1	4	3
SchWRKY070	g13833.t1	Chr08	31496223	32725386	+	344	WRKYGQK	C-X5-C-X23-H-X-H	1	3	2
SchWRKY071	g30258.t1	Chr10	4306090	4699578	-	291	WRKYGQK	C-X7-C-X23-H-X-C	1	3	2
SchWRKY072	g4219.t1	Chr01	52854705	55357085	+	345	WRKYGQK	C-X7-C-X23-H-X-C	1	3	2
SchWRKY073	g9466.t1	Chr03	33106883	35090941	+	354	WRKYGQK	C-X7-C-X23-H-X-C	1	3	2
SchWRKY074	g6934.t1	Chr05	36690180	38833165	+	293	WRKYGQK	C-X8-C-X24-H-X-C	1	3	2
SchWRKY075	g6933.t1	Chr05	36685259	38828822	+	332			1	3	2
SchWRKY076	g6930.t1	Chr05	36663079	38806170	-	286	WRKYGQK	C-X5-C-X23-H-X-C	1	3	2
SchWRKY077	g3563.t1	Chr06	18024538	21334726	-	224	WRKYGQK	C-X7-C-X23-H-X-C	1	3	2
SchWRKY078	g22449.t1	Chr03	25147172	25844998	-	278	WRKYGQK	C-X7-C-X24-H-X-H	1	3	2
SchWRKY079	g32230.t1	Chr09	11724081	12053169	-	294	WRKYGQK	C-X7-C-X23-H-X-C	1	3	2
SchWRKY080a	temp_model_12.1.57a38f05	Chr10	28897084	29758006	-	219	WRKYGQK	C-X7-C-X23-H-X-H	1	3	2
SchWRKY080b	novel_model_169_57a387f8	Chr10	28897084	29758006	-	215	WRKYGQK	C-X7-C-X23-H-X-H	1	3	2
SchWRKY081	g3537.t1	Chr06	17465475	20778400	+	365	WRKYGQK	C-X7-C-X23-H-X-H	1	4	3
SchWRKY082	g37078.t1	Chr01	47706248	47863155	-	250	WRKYGQK	C-X4-C-X23-H-X-H	1	4	3
SchWRKY083	g15604.t1	Chr05	29730094	30820242	-	265	none	C-X5-C-X23-H-X-H	1	2	1
SchWRKY084	-	-	-	-	-	298	WRKYGQK	C-X5-C-X23-H-X-H	1	2	1
SchWRKY084_like	-	-	-	-	-	180	WRKYGQK	C-X5-C-X23-H-X-H	1	2	1
SchWRKY085	g10699.t1	Chr09	1393671	2968615	+	304	WRKYGQK	C-X5-C-X23-H-X-H	1	3	2
SchWRKY086a	g9512.t1	Chr03	33672805	35657176	-	457	WRKYGQK	C-X6-C-X23-H-X-H	1	3	2
SchWRKY086b	g9513.t1	Chr03	33700699	35685074	-	458	WRKYGQK	C-X6-C-X23-H-X-H	1	3	2
SchWRKY088	g9374.t1	Chr03	32070423	34053848	-	217	WRK	C-X6-C-X23-H-X-H	1	1	0
SchWRKY089	g9372.t1	Chr03	32067604	34050774	-	132	none	C-X5-C-X23-H-X-H	1	1	0
